# Factors associated with reporting of the Prevention of Falls Network Europe (ProFaNE) core outcome set domains in randomized trials on falls in older people: a citation analysis and correlational study

**DOI:** 10.1186/s13063-022-06642-w

**Published:** 2022-08-26

**Authors:** Alexandra M. B. Korall, Dawn Steliga, Sarah E. Lamb, Stephen R. Lord, Rasheda Rabbani, Kathryn M. Sibley

**Affiliations:** 1 George & Fay Yee Centre for Healthcare Innovation, Third Floor, Chown Building, 753 McDermot Avenue, Winnipeg, MB R3B 0V8 Canada; 2 Department of Community Health Sciences, Rady Faculty of Health Sciences, Max Rady College of Medicine, University of Manitoba, Winnipeg, MB Canada; 3 Rady Faculty of Health Sciences, Interdisciplinary Health Program, University of Manitoba, Winnipeg, MB Canada; 4 College of Medicine and Health, University of Exeter, Exeter, UK; 5 Neuroscience Research Australia, New South Wales, Australia; 6 School of Public Health and Community Medicine, University of New South Wales, Sydney, NSW Australia

**Keywords:** Core outcome set, Adherence, Implementation fidelity, Accidental falls, Fall injuries, Psychological consequences of falls, Physical activity, Quality of life, Older people

## Abstract

**Background:**

Core outcome sets are advocated as a means to standardize outcome reporting across randomized controlled trials (RCTs) and reduce selective outcome reporting. In 2005, the Prevention of Falls Network Europe (ProFaNE) published a core outcome set identifying five domains that should be measured and reported, at a minimum, in RCTs or meta-analysis on falls in older people. As reporting of all five domains of the ProFaNE core outcome set has been minimal, we set out to investigate factors associated with reporting of the ProFaNE core outcome set domains in a purposeful sample of RCTs on falls in older people.

**Methods:**

We conducted a systematic citation analysis to identify all reports of RCTs focused on falls in older people that cited the ProFaNE core outcome set between October 2005 and July 2021. We abstracted author-level, study-level, and manuscript-level data and whether each domain of the ProFaNE core outcome set was reported. We used penalized LASSO regression to identify factors associated with the mean percentage of ProFaNE core outcome set domains reported.

**Results:**

We identified 85 eligible reports of RCTs. Articles were published between 2007 and 2021, described 75 unique RCTs, and were authored by 76 unique corresponding authors. The percentage of ProFaNE core outcome set domains reported ranged from 0 to 100%, with a median of 40% and mean (standard deviation, SD) of 52.2% (25.1). RCTs funded by a non-industry source reported a higher mean percentage of domains than RCTs without a non-industry funding source (estimated mean difference = 17.5%; 95% confidence interval (CI) 1.8–33.2). RCTs examining exercise (15.4%; 95% CI 1.9–28.9) or multi-component/factorial (17.4%; 95% CI 4.7–30.1) interventions each reported a higher mean percentage of domains than RCTs examining other intervention types.

**Conclusions:**

We found that RCTs funded by at least one non-industry source, examining exercise or multi-component/factorial interventions, reported the highest percentages of ProFaNE core outcome set domains. Findings may help inform strategies to increase the impact of the ProFaNE core outcome set. Ultimately, this may lead to enhanced knowledge of the effectiveness and safety of interventions to prevent and/or manage falls in older people.

**Supplementary Information:**

The online version contains supplementary material available at 10.1186/s13063-022-06642-w.

## Background

About 20–30% of community-dwelling people over the age of 65 fall annually [1–3]. Falls are the leading cause of injury-related hospitalizations and deaths in older people [4–6]. Serious injuries arising from falls include cuts or lacerations, soft tissue injuries, fractures, and traumatic brain injuries [7–9]. Even falls that do not result in physical harm may have debilitating effects on an older person’s health and quality of life, by contributing to fear of falling, activity restriction, reduced social participation, and depressive symptoms [10–15]. In addition to physical and psychological sequelae, the economic costs of falls are substantial. In Canada, falls cost an estimated $8 million annually [4]; in the United Kingdom (UK), the cost of falls in older people was estimated at £981 million in 1999 [16]; and in the United States (US), medical costs attributable to falls were estimated to be near $50 billion in 2015 [17].

The clinical effectiveness of interventions to prevent falls and their debilitating effects is best measured using systematic reviews and meta-analyses of quasi-randomized or randomized controlled trials (RCTs), as they yield the most precise effect estimates, optimize statistical power, and can measure and examine reasons for heterogeneity across studies [18]. Although there is strong evidence that exercise is effective at preventing falls in community-dwelling older adults (e.g. [19, 20]), we have limited understanding of the effects of many interventions on rates of serious injury and overall quality of life [20]. This is partly due to inconsistent outcome reporting across RCTs, as the body of evidence may be too small or cannot be synthesized when outcomes are not evaluated consistently. For example, in a recent systematic review and network meta-analysis examining fall-prevention interventions for adults aged 65 years or older in all settings, less than a quarter of eligible RCTs were included in network meta-analyses on injurious falls (54 of 283 RCTs, 19%), fractures (68 of 283 RCTs, 24%), and hip fractures (39 of 283 RCTs, 14%), while fewer than 1% of eligible RCTs were included in a network meta-analysis on quality of life (2 of 283 RCTs) [20]. Additionally, a high percentage of RCTs (183 of 283 RCTs, 65%) had an unclear risk of bias for selective outcome reporting [20], which threatens the validity of existing and future systematic reviews and meta-analysis, and can undermine our understanding of the true effectiveness and safety of interventions [21].

A core outcome set (COS) is an agreed standardized collection of outcomes that should be measured and reported, at a minimum, in a specific area of health [22, 23]. COS are advocated as means to standardize outcome reporting across trials, facilitate knowledge synthesis, minimize the risk of bias from selective outcome reporting, ensure important outcomes are measured and reported, and consequently reduce research waste and increase the impact and benefits of research [24–27]. COS often include outcome measurement instruments, which provide details on how to measure and report each outcome included in the COS [28].

In 2005, Lamb and colleagues, on behalf of the Prevention of Falls Network Europe (ProFaNE), published a COS identifying five core domains that should be measured and reported, at a minimum, in future randomized trials and meta-analysis focused on falls in community-dwelling older people: falls, injuries, psychological consequences of falling, generic health-related quality of life (HRQoL), and physical activity [29]. The ProFaNE group explained that “the desirable profile of an intervention is one that improves activity and confidence while reducing falling. HRQoL is likely to capture unanticipated effects of an intervention in terms of general, emotional, and social health.” (p.1619) [29].

Awareness of the ProFaNE COS is high, with more than 800 citations by researchers spanning multiple countries. Even so, reporting of all five domains of the ProFaNE COS has been minimal in RCTs examining the effectiveness or safety of interventions to prevent and/or manage falls in older people [30]. In the first decade after its publication, the ProFaNE COS was cited in 34 peer-reviewed articles reporting the results of RCTs focused on falls in older people [30]. Of these, one (3%) reported the effects of an intervention on all five domains of the ProFaNE COS [30]. Falls were most frequently reported (32 of 34, 94%), followed by physical injuries (16 of 34, 47%), while psychological consequences of falling (7 of 34, 21%), physical activity (8 of 34, 24%), and HRQoL (8 of 34, 24%) were each reported in less than a quarter of RCTs [30].

Several studies have been conducted on the impact of COS [31–34]. Most research on the implementation of COS has focused on understanding barriers and facilitators to their *adoption* (i.e. uptake), defined as the initial intention or decision to try to employ an innovation or evidence-based practice [35]. Hughes et al. surveyed chief investigators of funded and non-funded applications submitted to the United Kingdom (UK) National Institute for Health Research (NIHR) Health Technology Assessment (HTA) Programme to investigate their reasons for deciding whether or not to include a COS in their proposals for a RCT [31]. Reasons cited by chief investigators for deciding to include a COS were the belief in the benefits of measuring a COS, having previous experience with the development of COS, and journal publication requirements [31]. Reasons cited for not including a COS in their proposals were being aware of conflicting guidance from granting agencies and patient and public partners, and a perceived need to measure and report the same outcomes as past trials, when the COS did not include those outcomes [31]. Wallace et al. surveyed trialists to understand their reasons for use and non-use of the Research Outcome Measurement in Aphasia (ROMA) COS, who identified belief in the benefits of using the ROMA COS as a facilitator, and lack of external incentives, collegial encouragement, and monitoring systems as barriers to using the ROMA COS [34]. Similarly, Fletcher et al. surveyed trialists to identify barriers to adoption of the hip fracture COS [33]. Key reasons for non-adoption included lack of awareness of the hip fracture COS during trial design and perceived inappropriateness of the COS for specific populations and intervention types [33].

Less attention has been paid to investigating factors influencing the *fidelity* of COS implementation, defined as the degree to which an intervention was implemented as it was prescribed in the original protocol or as it was intended by the programme developers [35]. Implementation fidelity has been conceptualized in terms of adherence to the programme protocol, dose or amount of the programme delivered, and quality of the programme delivered [35]. In other words, COS must first be adopted and then implemented to some degree of fidelity. A better understanding of factors influencing both the adoption (i.e. uptake) and fidelity of implementation (i.e. adherence) of COS is needed to develop and tailor strategies to realize their impact.

Despite this need, we have limited understanding of barriers and enablers to implementation of the ProFaNE COS. As we understand it, no research has investigated factors governing the extent to which the ProFaNE COS has been implemented with fidelity in RCTs focused on falls in older people. This knowledge may help inform strategies to increase the impact of the ProFaNE COS, which in turn should lead to more consistent outcome reporting across RCTs and, ultimately, better understanding of the effectiveness and safety of interventions to prevent and/or manage falls in older people. Thus, by way of a systematic citation analysis and correlational study in a purposeful sample of RCTs examining the safety or effectiveness of interventions to prevent and/or manage falls in older people, our objectives are twofold: (i) to identify factors associated with the percentage of ProFaNE COS domains reported and (ii) to identify factors associated with the likelihood of reporting each domain of the ProFaNE COS.

## Methods

### Study design

We conducted a systematic citation analysis of the ProFaNE COS and correlational study to identify factors associated with reporting of the ProFaNE COS in a purposeful sample of RCTs examining the safety or effectiveness of interventions to prevent and/or manage falls in older people.

### Theoretical underpinning

Our investigation into factors associated with reporting of ProFaNE COS domains was informed by the Consolidated Framework for Implementation Research (CFIR) [36]. Some associations were hypothesis-driven; however, as little is known about factors influencing reporting of COS in RCTs, others were driven by exploration, but based on the assumption that characteristics of the inner setting, outer setting, intervention, and individuals involved are possible determinants of knowledge use [36].

### Outcomes

Our primary outcome was the percentage of ProFaNE COS domains reported. Secondary outcomes included whether falls were reported (yes, no), injuries were reported (yes, no), psychological consequences of falling were reported (yes, no), HRQoL was reported (yes, no), and physical activity was reported (yes, no).

### Explanatory variables and study hypotheses

We examined associations between author-level, study-level, and manuscript-level factors and primary and secondary outcomes.

#### Author-level variables

We included a single author-level variable—whether the geographic affiliation of the corresponding author was European vs. other. The ProFaNE COS was developed on behalf of a European network [29]. The CFIR states that the perception of stakeholders about whether the intervention or evidence-informed practice (here, the COS) is internally or externally developed can influence implementation success [36]. It is possible investigators within Europe may be more likely to perceive the ProFaNE COS was internally developed than investigators outside Europe. We hypothesized that articles authored by a corresponding author affiliated with one or more European institutions would report a greater percentage of ProFaNE COS domains than articles authored by a corresponding author based outside of Europe.

#### Study-level variables

Study-level variables included the setting (community vs. institution or combined), number of trial centres (single, multiple, not reported), number of trial arms (two vs. three or more), funding source(s) (at least one source of non-industry vs. no non-industry source(s) or not reported/unclear), intervention type (exercise, multi-component/factorial, other), mean age of the sample at baseline, percentage of the sample that was female at baseline, whether the population of interest had a specific disease diagnosis (yes, no), fall risk of participants at baseline (at risk, at high risk), sample size, and length of follow-up of the primary outcome variable (12 months or more vs. less than 12 months).

The CFIR states that the compatibility of the intervention or evidence-informed practice (the COS) with the local context, or inner setting, may influence the success of implementation [36]. Lamb et al. specified the selection of outcomes in the ProFaNE COS should focus on community-dwelling populations [29]. We hypothesized that articles reporting the results of RCTs conducted in community settings would report a greater percentage of domains than articles reporting the results of RCTs set in institutions, such as hospitals, long-term care or nursing homes, and assisted-living facilities.

Outcomes included in the ProFaNE COS are generally measured by self-report and were selected to accommodate “nearly all community-dwelling older people” [29]. Copsey et al. reported that some RCTs citing the ProFaNE COS restricted their population of interest to people with dementia or cognitive impairment, Parkinson’s disease, or stroke [30]. The measurement and validity of some or all of these outcomes in persons living with specific disease diagnoses, especially cognitive impairment or dementia [37], may require additional considerations when compared to the general population of community-dwelling older adults. Previously, in an international survey of trialists on their reasons for non-adoption of the hip fracture COS, more than half (54%) of respondents stated the COS was not appropriate for trials on people living with cognitive impairment and needed revision to accommodate this population [33]. We hypothesized that articles reporting the results of RCTs in populations with specific disease diagnoses, including but not limited to cognitive impairment or dementia, would report a smaller percentage of ProFaNE COS domains than articles reporting the results of RCTs in a general population of older adults.

#### Manuscript-level variables

Manuscript-level variables included the year of publication, the type of journal (specialist vs. general), and whether the manuscript cited the ProFaNE COS in the introduction (yes, no), methods (yes, no), and discussion (yes, no) sections. We hypothesized that articles citing the ProFaNE COS in the methods section would report a greater percentage of domains than articles that did not cite the ProFaNE COS in the methods section.

### Population of interest and eligibility criteria

Our aim was to investigate factors governing the extent to which the ProFaNE COS was implemented with fidelity as intended by developers. Consistent with this aim, we made several decisions to restrict our population of interest. First, we only included articles citing the ProFaNE COS, as we interpreted citation as evidence of adoption (i.e. uptake). To be implemented with fidelity requires that an innovation or evidence-based practice must first be adopted by an individual, organization, or setting [35]. Second, we restricted our sample to completed RCTs, defined as prospective studies that assessed healthcare interventions in human participants who were randomly allocated to study groups [30]. As stated by developers, the ProFaNE COS was “intended to promote consistency in collection and reporting of essential elements (page 1619)” in future trials and meta-analyses [29]. We excluded non-completed RCTs, such as protocols, due to the prevalence of outcome modification after trial initiation and selective outcome reporting (e.g. [38]), making it impossible to explore implementation fidelity based on reporting of pre-specified outcomes. We also excluded early developmental trials, such as feasibility and pilot studies, where the focus was to examine whether the trial should be done, and if so, how. Third, we restricted our sample to RCTs that sampled older people, defined as whether the study excluded participants younger than 60 years of age, the mean age of included participants was 60 years of age or above, or the patient population was described as ‘older’, ‘elderly’, or ‘senior’ [30]. Fourth, we restricted our sample to RCTs examining the safety, effectiveness, or cost-effectiveness of interventions to prevent and/or manage falls. Fifth, we excluded articles if they were published in a language other than English. This was a practical consideration as time and funding were limited. Last, when more than one article was published on the results of a single RCT, we included all articles if they reported different outcomes at the same follow-up point analysed after trial completion, included the first article (determined by the date of publication) only when they reported the same outcomes at the same follow-up points analysed after trial completion, and included all articles if they reported the same or different outcomes at different follow-up points analysed after trial completion. This provided insight into temporal changes in barriers and enablers to the reporting of ProFaNE COS domains over the life course of a given trial.

### Data sources

To identify eligible articles, we conducted a systematic citation analysis of the ProFaNE COS between 01 October 2005 and 12 July 2021. However, our search strategy differed for articles published prior to and after 16 January 2015. As Copsey et al. previously conducted a systematic citation analysis for all articles citing the ProFaNE COS between 01 October 2005 and 16 January 2015, we screened their list of included RCTs to identify articles published prior to 17 January 2015 [30]. As our eligibility criteria only differed from Copsey et al.’s with respect to the inclusion of multiple articles reporting data from the same RCT, we also screened Copsey et al.’s [30] list of RCTs excluded because they were secondary reports of already included RCTs. To identify articles citing the ProFaNE COS after 17 January 2015 and before 12 July 2021, we searched the Web of Science Citation Index, PubMed, and Scopus databases.

### Article selection

To screen articles for eligibility, we adopted a two-step approach involving the title and abstract screening followed by full-text screening. To facilitate high-quality screening, reviewers were provided with protocols for screening. All difficulties, and their associated resolutions, were logged on screening tracking forms.

Our screening process differed for articles published prior to and after 17 January 2015. A single reviewer (AMBK) independently screened the titles and abstracts and subsequently the full text of articles published prior to 17 January 2015 for eligibility. We limited screening to a single reviewer as all articles published prior to 17 January 2015 had previously undergone screening by Copsey et al. [30]. Comparatively, at least two reviewers independently screened the titles and abstracts and subsequently the full text of articles published after 17 January 2015 for eligibility, as per the following. First, after removing duplicates, at least two reviewers independently screened the title and abstract of each article to determine the study type. Then, at least two reviewers independently screened the full text of the subset of articles with study types coded as RCT and unsure. When necessary, a third reviewer screened articles with conflicts between first and second screenings to resolve conflicts. If the third reviewer disagreed with both the first and second reviewer, two reviewers (AMBK and KMS) met in person to discuss and resolve conflicts.

### Data abstraction

At least two reviewers independently abstracted data on whether each domain of the ProFaNE COS was reported, as well as author-level, study-level, and manuscript-level data from all articles meeting our eligibility criteria. To do this, a single reviewer (DS) from our research team first independently abstracted data from articles included in the analysis by Copsey et al., which also fulfilled our eligibility criteria [30]. Upon request, Copsey et al. shared the data they abstracted from these articles, which constituted the second abstraction, where possible [30]. For variables not abstracted by Copsey et al. [30], a second reviewer from our research team (AMBK) abstracted data from this subset of articles. Then, four reviewers, including one member of the research team (DS) and three research assistants, independently abstracted data from the remaining articles included in our sample. Reviewers were provided with a protocol for data abstraction and were instructed to log all difficulties and their associated resolutions on data abstraction tracking forms. A third, independent reviewer (AMBK or KMS) identified and resolved conflicts between the first and second abstractions.

### Statistical analysis

Statistical significance in the final models was defined as *P* < 0.05. For all analyses, SAS (9.4) software, Cary, NC, US, was used.

#### Primary outcome: percentage of ProFaNE COS domains reported

We used the penalized regression method to select important predictors. This method allowed us to create a linear regression model that is penalized, for having too many variables in the model, by adding a constraint in the Eq. [39]. We used the LASSO (Least Absolute Shrinkage and Selection Operator) method [40] using the GLMSELECT procedure in SAS [41, 42], whereby a sequence of models is obtained using the LASSO algorithm, and in this sequence, the one yielding the smallest value of the Mallow’s C(p) statistic [43] is chosen as the final model. We produced diagnostic plots to select the most parsimonious model (Additional file 1). We used the split option for categorical variables to include all levels as dummy coded variables in the model. In the LASSO selection procedure, it is not possible to produce *P* values for the selected variables. In lieu, we ran a generalized linear model with these selected variables to estimate the mean difference and its 95% confidence intervals (95% CI) and *P* values to describe the variables that have significant relationships with the outcome, as that was our main interest.

#### Secondary outcomes: reporting of each domain of the ProFaNE COS

We used statistical models for classification to select important variables that are useful for predicting the outcome variable. We used cost-complexity pruning [44] and estimated the misclassification rate by tenfold cross-validation to prevent overfitting. For all models, the fitted model classified the response variable well (Additional file 2). The model is estimated using the HPSPLIT procedure in SAS [42]. In addition, we ran a generalized linear model with those selected variables to estimate the odds ratio (OR) and its 95% CI and *P* values to describe the variables that have significant relationships with each outcome, as that was our main interest.

#### Missing data

We were missing data for three explanatory variables and no outcome variables. We could not determine the number of trial centres in *n* = 13 (15.3%) articles, funding type in *n* = 4 (4.7%) articles, and length of follow-up for the primary outcome variable in *n* = 1 (1.2%) article. We used different approaches to handle missingness in our statistical analyses. First, we list-wise deleted the single article containing missing data for the length of follow-up of the primary outcome variable. Then, to treat missing data on the number of trial centres and funding type, we created missing data response options (“Not Reported”) for these variables. We entered these response options in statistical models either as their own category (number of trial centres) when frequencies permitted, or as part of an ‘Else’ category where missing cases were grouped with other response options (funding type). Thus, our final analytical sample comprised *n* = 84 articles.

## Results

### Data sources

By scanning the lists of RCTs included in and excluded from the analysis by Copsey et al. [30], we identified *n* = 44 unique articles that cited the ProFaNE COS and were published prior to 17 January 2015. Through database searching, we identified *n* = 761 unique articles that cited the ProFaNE COS and were published after 17 January 2015 and before 12 July 2021.

### Article selection

**Fig. 1:**
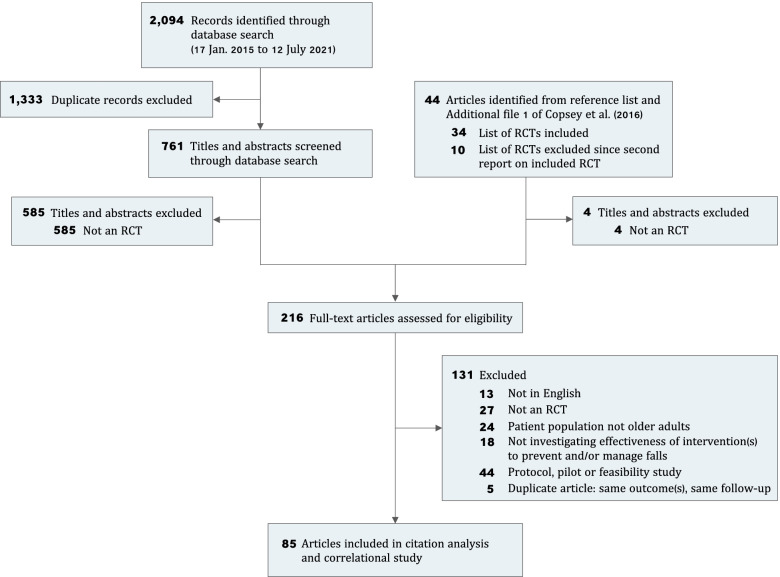
Article flow from citation analysis. RCT indicates randomized controlled trial

We screened a total of *n* = 805 titles and abstracts and subsequently the full text of *n* = 216 articles (Fig. 1). Of the *n* = 216 articles that underwent full-text review, we included *n* = 85 articles (Additional file 3) and excluded *n* = 131 articles (Additional file 4).

### Characteristics of included articles

Articles were most often authored by corresponding authors affiliated with institutions from Australia (18 of 85, 21%), followed by the UK (12 of 85, 14%), Germany (10 of 85, 12%), US (9 of 85, 11%), and Finland (6 of 85, 7%) (Table 1). The majority of articles (49 of 85, 58%) were authored by corresponding authors affiliated with one or more European institutions at the time of publication. Articles were authored by 76 unique corresponding authors.

**Table 1:** Characteristics of the included articles (*n* = 85)

Characteristic	
***Author-level variables***	
Affiliation of the corresponding author^a^	
European institute, no. (%)	49 (57.6)
Finland, no. (%)	6 (7.1)
Germany, no. (%)	10 (11.8)
Spain, no. (%)	3 (3.5)
Sweden, no. (%)	4 (4.7)
Switzerland, no. (%)	3 (3.5)
The Netherlands, no. (%)	4 (4.7)
UK, no. (%)	12 (14.1)
Non-European institute, no. (%)	36 (42.4)
Australia, no. (%)	18 (21.2)
New Zealand, no. (%)	4 (4.7)
USA, no. (%)	9 (10.6)
***Study-level variables***	
Setting	
Community, no. (%)	69 (81.2)
Other, no. (%)	16 (18.8)
Assisted living, no. (%)	1 (1.2)
Hospital, no. (%)	5 (5.9)
Long-term care/nursing home, no. (%)	6 (7.1)
Combined, no. (%)	4 (4.7)
Number of trial centres	
Multiple, no. (%)	53 (62.4)
Single, no. (%)	19 (22.4)
Not reported, no. (%)	13 (15.3)
Number of trial arms	
Two, no. (%)	67 (78.8)
Three or more, no. (%)	18 (21.2)
Funding source	
Industry, no. (%)	3 (3.5)
Non-industry, no. (%)	63 (74.1)
Combined, no. (%)	12 (14.1)
None, no. (%)	3 (3.5)
Not reported or unclear, no. (%)	4 (4.7)
Intervention type	
Exercise, no. (%)	26 (30.6)
Multi-component/factorial, no. (%)	38 (44.7)
Other, no. (%)	21 (24.7)
Mean age of trial participants at baseline	
Years, mean (SD)	76.8 (5.3)
Years, minimum	62
Years, maximum	88
Female participants at baseline	
%, mean (SD)	68.1 (17.8)
%, minimum	0
%, maximum	100
Whether population of interest living with disease at baseline	
No, no. (%)	61 (71.8)
Yes, no. (%)	24 (28.2)
Dementia or cognitive impairment, no. (%)	6 (7.0)
Parkinson’s disease and/or stroke, no. (%)	5 (5.9)
Other, no. (%)	13 (15.3)
Fall risk of trial participants at baseline	
At high risk^b^, no. (%)	49 (57.6)
At risk, no. (%)	36 (42.4)
Sample size at baseline	
Mean (SD)	837.2 (2029.3)
Minimum	23
Maximum	12,483
Length of follow-up of primary outcome	
Less than 12 months, no. (%)	32 (37.6)
12 or more months, no. (%)	52 (61.2)
Not reported, no. (%)	1 (1.2)
***Manuscript-level variables***	
Year of publication	
2007–2011, no. (%)	22 (25.9)
2012–2016, no. (%)	30 (35.3)
2017–2021, no. (%)	33 (38.5)
Journal type	
Specialist, no. (%)	62 (72.9)
General, no. (%)	23 (27.1)
Citation of ProFaNE core outcome set	
Introduction, no. (%)	12 (14.1)
Methods, no. (%)	72 (84.7)
Results, no. (%)	1 (1.2)
Discussion, no. (%)	17 (20.0)

*SD* standard deviation

^a^ *n* = 3 articles had corresponding authors with affiliations from multiple countries

^b^ if a history of falls was included in eligibility criteria, or participants were described as being at high risk for falls

Most (69 of 85, 81%) articles reported the results of RCTs based in community settings, 75 (of 85, 88%) reported at least one non-industry source of funding, and 24 (of 85, 28%) reported the results of RCTs in populations with specific disease diagnoses, including dementia or cognitive impairment (6 of 85, 7%), Parkinson’s and/or stroke (5 of 85, 6%), and others (13 of 85, 15%) (Table 1). Articles reported the results of 75 unique RCTs.

Articles were published between 2007 and 2021, with the largest proportion published after 2017 (33 of 85, 39%) (Table 1). The ProFaNE COS was cited in the introduction section by *n* = 12 (of 85, 14%) articles, including when providing the rationale for the article by justifying their selection, measurement and reporting of outcomes (*n* = 7), when providing a definition of a ‘fall’ (*n* = 3), and when synthesizing the evidence of the effectiveness of interventions to prevent falls or recommendations for clinical practice (*n* = 2). The ProFaNE COS was cited in the methods section by *n* = 72 (of 85, 85%) articles, namely when describing measurement and/or reporting of the falls domain (*n* = 69), including when providing a definition of a ‘fall’ (*n* = 52). Seven, three, two, and one articles cited the ProFaNE COS in the methods section when describing measurement and/or reporting of injuries, HRQoL, psychological consequences of falling, and physical activity, respectively. The ProFaNE COS was cited in the discussion section by *n* = 17 (of 85, 20%) articles, when discussing the strengths of the study afforded by following recommendations from the ProFaNE COS (*n* = 9), limitations of the study afforded by not following recommendations from the ProFaNE COS (*n* = 5), potential shortcomings of measuring falls via self-report (*n* = 1), issues related to the definition of falls provided in the COS (*n* = 1), and considerations made when selecting an instrument to measure HRQoL (*n* = 1).

### Reporting of ProFaNE COS domains

The percentage of domains of the ProFaNE COS reported ranged from 0 to 100% across articles, with a median of 40% and a mean (SD) of 52.2% (25.1). Five domains were reported in *n* = 8 (of 85, 9%) articles, four domains in *n* = 12 (of 85, 14%) articles, three domains in *n* = 22 (of 85, 26%) articles, two domains in *n* = 27 (of 85, 32%) articles, one domain in *n* = 14 (of 85, 16%) articles, and zero domains in *n* = 2 (of 85, 2%) articles. Of the two articles that reported zero domains, one reported the effects of education on older adults’ knowledge of fall threats and fall-prevention behaviours [44] and the other reported the effects of community-based exercise programmes on the following risk factors for falls: balance, postural control, mobility, and leg strength [45]. The former purported to measure falls, but did not report any data on falls [44]. Of the ProFaNE COS domains, falls were reported most frequently (76 of 85, 89%), followed by injuries (47 of 85, 55%), psychological consequences of falling (39 of 85, 46%), and physical activity (31 of 85, 37%), while HRQoL was reported least often (29 of 85, 31%) (Table 2).

**Table 2:** Number (percentage) of articles (*n* = 85) reporting each domain of the Prevention of Falls Network Europe (ProFaNE) core outcome set

ProFaNE core outcome set domain	No. (%)
Falls	76 (89)
Injuries	47 (55)
Psychological consequences of falls	39 (46)
Health-related quality of life	29 (31)
Physical activity	31 (37)

### Factors associated with reporting of ProFaNE COS domains

#### Overall percentage of ProFaNE COS domains reported

Funding type and intervention type were associated with the percentage of ProFaNE COS domains reported (Table 3). In terms of funding type, articles that reported at least one non-industry source of funding reported a higher mean percentage of domains from the ProFaNE COS than articles that did not report at least one non-industry source of funding (estimated mean difference = 17.5%; 95% CI 1.2–33.2). In terms of intervention type, RCTs that tested exercise (estimated mean difference = 15.4%; 95% CI 1.9–28.9) or multi-component/factorial (estimated mean difference = 17.4%; 95% CI 4.7–30.1) interventions each reported a higher mean percentage of domains from the ProFaNE COS when compared to RCTs that examined other interventions.

**Table 3:** Explanatory variables associated with the percentage of the Prevention of Falls Network Europe (ProFaNE) core outcome set domains reported

Variable	Mean difference	95% CI	*P* value
Funding type			
Non-industry or combined (*n* = 74) vs. else (*n* = 10)	17.5	1.8–33.2	.03
Intervention type			
Exercise (*n* = 25) vs. other^a^ (*n* = 21)	15.4	1.9–28.9	.03
Multi-component/multi-factorial (*n* = 38) vs. other^a^ (*n* = 21)	17.4	4.7–30.1	.01

^a^Includes advice (*n* = 6), drug or supplement (*n* = 3), environment or aide (*n* = 3), medical (*n* = 2), or other intervention types (*n* = 7)

#### Reporting of falls

The length of follow-up of the primary outcome variable and citation of the ProFaNE COS in the methods section were associated with the likelihood of reporting falls (Table 4.). The odds of reporting falls was higher in articles that reported primary outcomes after at least 12 months of follow-up compared to less than 12 months of follow-up (OR = 8.2; 95% CI 1.7–65.2). The odds of reporting falls was lower in articles that did not cite the ProFaNE COS in the methods section when compared to articles that did (OR = 0.02; 95% CI < 0.01–0.12).

**Table 4:** Explanatory variables associated with the likelihood of reporting each domain of the Prevention of Falls Network Europe (ProFaNE) core outcome set

ProFaNE core outcome set domain	Odds ratio	95% CI	*P* value
**Falls**			
Cited methods			
No (*n* = 71) vs. yes (*n* = 13)	0.02	< 0.01–0.12	< .01
1° outcome follow-up			
≥ 12 months (*n* = 52) vs. < 12 months (*n* = 32)	8.2	1.7–65.2	.02
**Injuries**			
1° outcome follow-up			
≥ 12 months (*n* = 52) vs. < 12 months (*n* = 32)	9.8	3.1–35.7	< .01
**Psychological consequences of falling**			
Intervention type			
Exercise (*n* = 25) vs. other^a^ (*n* = 21)	3.8	0.8–20.8	.11
Multi-component/factorial (*n* = 38) vs. other^a^ (*n* = 21)	8.8	1.9–51.4	.01
Cited discussion			
No (*n* = 67) vs. yes (*n* = 17)	6.2	1.7–35.6	.03
**Health-related quality of life**			
Number of trial arms			
Three or more (*n* = 18) vs. two (*n* = 66)	0.08	< 0.01–0.54	.04
**Physical activity**			
Intervention type			
Exercise (*n* = 25) vs. other^a^ (*n* = 21)	25.3	3.6–303.6	< .01
Multi-component/factorial (*n* = 38) vs. other^a^ (*n* = 21)	7.5	1.3–76.0	.04

^a^ Includes advice (*n* = 6), drug or supplement (*n* = 3), environment or aide (*n* = 3), medical (*n* = 2), or other intervention types (*n* = 7)

#### Reporting of injuries

The length of follow-up of the primary outcome variable was associated with the likelihood of reporting injuries (Table 4). The odds of reporting injuries was higher in articles that reported primary outcomes after at least 12 months of follow-up when compared to less than 12 months of follow-up (OR = 9.8; 95% CI 3.1–35.7).

#### Reporting of psychological consequences of falls

Intervention type and citation of the ProFaNE COS in the discussion section were associated with the likelihood of reporting psychological consequences of falling (Table 4). The odds of reporting psychological consequences of falls was higher in RCTs that tested multi-component/factorial interventions when compared to other intervention types that did not involve exercise (OR = 8.8; 95% CI 1.9–51.4). The odds of reporting psychological consequences of falls was higher in articles that did not cite the ProFaNE COS in the discussion section when compared to articles that did (OR = 6.1; 95% CI 1.7–35.6).

#### Reporting of HRQoL

The number of experimental arms was associated with the likelihood of reporting HRQoL (Table 4). The odds of reporting HRQoL was lower in RCTs with multiple experimental arms compared to a single experimental arm (OR = 0.08; 95% CI < 0.01–0.54).

#### Reporting of physical activity

The intervention type was associated with the likelihood of reporting physical activity (Table 4). The odds of reporting physical activity was higher in RCTs that tested exercise (OR = 25.3; 95% CI 3.6–303.6) and multi-component/factorial (OR = 7.5; 95% CI 1.3–76.0) interventions when compared to other intervention types.

## Discussion

By way of a systematic citation analysis and correlational study, we aimed to identify factors associated with reporting of the ProFaNE COS in a purposeful sample of articles disseminating the results of RCTs focused on falls in older people, which cited the ProFaNE COS.

Consistent with findings from Copsey et al. [30], we observed that the number of articles citing the ProFaNE COS has increased over time. Between January 2015 and July 2021, the ProFaNE COS was cited by 761 unique articles. Previously, Copsey et al. reported that between October 2005 and January 2015, the ProFaNE COS was cited by 464 unique articles [30]. Our findings lend support to Copsey et al.’s [30] claim that awareness of the ProFaNE COS has increased, and is likely to continue to increase, over time [30].

We also found that few of the included trials implemented the ProFaNE COS with fidelity. Just eight articles (of 85, 9%) reported all five domains of the ProFaNE COS, and most measured two (27 of 85, 32%). Additionally, as was observed by Copsey et al., not all domains of the ProFaNE COS were reported equally [30]. Falls were reported most often (76 of 85, 89%), followed by injuries (47 of 85, 55%), psychological consequences of falling (39 of 85, 46%), physical activity (31 of 85, 37%), and HRQoL (29 of 85, 31%).

The limited impact of COS has been reported previously. Smith et al. examined the impact of the hip fracture COS in RCTs registered in the ClinicalTrials.gov trial registry between 1997 and 2018 [32]. Of these, *n* = 96 RCTs fitted with the scope of the COS and were registered between 2015 and 2018, i.e. after dissemination of the hip fracture COS in 2014. Only 7 (7%) trials included all five domains of the hip fracture COS. Mortality, pain, activities of daily living, mobility, and HRQoL were included in 41% (39 of 96), 43% (41 of 96), 32% (31 of 96), 43% (41 of 96), and 25% (24 of 96) of registered trials, respectively [32].

Regarding our primary objective, we observed that funding type was associated with the percentage of ProFaNE COS domains reported. RCTs with at least one non-industry funding source reported a higher mean percentage of domains than RCTs without at least one non-industry funding source. This observation may, in part, reflect initiatives of non-industry trial funders across Europe (e.g. Horizons2020) and the US (e.g. Patient-Centered Outcomes Research Institute; PCORI) to endorse COS, often by requiring trialists to search for relevant COS, and to justify when relevant COS are not included in proposals for clinical trials. For example, in 2012, the UK NIHR HTA Programme directed applicants for all randomized trial and evidence synthesis funding streams to the Core Outcome Measures in Effectiveness Trials (COMET) database of planned, completed, and ongoing COS (www.comet-initiative.org), stating that “where established Core Outcomes exist they should be included amongst the list of outcomes unless there is good reason to do otherwise”. An evaluation of the extent to which applicants followed these guidelines and searched for a relevant COS between 2012 and 2015 concluded that this endorsement had a positive impact on COS uptake [31]. Findings may also be attributable to the nature of ProFaNE COS domains. Extant research suggests that for-profit-funded trials report favourable (positive) outcomes more often than trials funded by other sources for several reasons, including greater reliance on surrogate endpoints and insufficient follow-up [45]. No surrogate endpoints were included in the ProFaNE COS, and the recommended length of follow-up for each domain was a minimum of 12 months. In future research, it might be informative to explore whether differences in reporting of ProFaNE COS domains exist between RCTs funded by government grants vs. other forms of non-industry funding. Trials funded by government grants that have undergone multiple stages of peer/panel review, with external steering committees monitoring methodology along the various stages of the process, might be expected to produce higher quality trials that are the most likely to implement COS with fidelity.

We observed that intervention type was associated with the percentage of ProFaNE COS domains reported, suggesting the appropriateness of the ProFaNE COS may be affected by the characteristics of the intervention under study. Findings are consistent with the results of an international survey of trialists on their reasons for non-adoption of a universal hip fracture COS developed to cover a broad scope of interventions, whereby 35% (28 of 80), 34% (27 of 80), and 24% (19 of 80) of respondents believed the existing COS needed to be revised to accommodate rehabilitation, surgical, and anaesthetic interventions, respectively [33].

Specifically, RCTs examining exercise or multi-component/factorial interventions each reported a higher mean percentage of domains compared to RCTs examining other intervention types, such as advice, environmental modification or aides, medical interventions, and drugs or supplements. In secondary analyses, we found that odds of reporting physical activity was higher in RCTs examining exercise or multi-component/factorial interventions, while odds of reporting psychological consequences of falls was higher in RCTs testing multi-component/factorial interventions, when compared to other intervention types. The reasons why multi-component/factorial and exercise intervention trials may have been more compatible with the ProFaNE COS remain unclear. A better understanding of the mechanisms by which intervention type could affect the compatibility of ProFaNE COS would be useful to design and tailor implementation strategies to improve the appropriateness of the ProFaNE COS for a diversity of healthcare interventions. One avenue of future research may investigate whether differences in the shared knowledge, beliefs, self-efficacy, and/or motivation of trialists testing different intervention types to measure and report ProFaNE COS domains could explain our findings, as these are theorized to influence implementation success [36].

Best practices for COS development recommend the scope of healthcare interventions (e.g. surgery, drugs, medical devices) covered by the COS should be defined to reduce ambiguity and help users decide on the relevance of the COS to their work [46]. Although not explicitly defined, the ProFaNE COS could be interpreted as applying to a broad scope of interventions. Challenges in implementing COS with universal population and intervention coverage have been reported before (e.g. hip fracture COS [33]). Future research may seek to explore whether implementation fidelity of COS is impacted by the broadness of scope, defined in terms of the research or practice setting covered, health conditions covered, populations covered, and interventions covered [46]. COS developers should strike to achieve an optimal balance between the generalizability and specificity of a COS. If defined too broadly in scope, the COS may be perceived as needing adaptation to fit heterogenous trial designs. Conversely, if defined too narrowly in scope, developers risk the COS having limited potential for impact.

It is worth noting that since the original publication of the ProFaNE COS in 2005, minimum standards have been set for developing COS [46]. At a minimum, it is recommended that COS should be developed in partnership with (1) individuals (or groups) who will use the COS in research, including clinical trialists and industry; (2) healthcare professionals; and (3) people with lived experience of the health condition, including patients, family members, and carers. It is also recommended that care should be taken to avoid ambiguity in the language used in the list of outcomes [46]. People with lived experience of falls did not contribute to the development of the ProFaNE COS, and of the five ProFaNE COS domains, two (physical activity and HRQoL) were undefined, and one (physical activity) was not accompanied by a recommended measurement instrument set. It was stated that “further research is required before a measure of physical activity can be recommended for inclusion in the common data set (page 1621)” [29]. It is beyond the scope of this study to understand if and how these limitations of the ProFaNE COS could have impacted implementation fidelity. Nevertheless, the ProFaNE COS may need updating to reflect current standards in COS development.

There are diverse methodological approaches to assess barriers and enablers to knowledge use, and none is considered superior to others [47]. We elected to use a quantitative approach by examining associations between potential determinants of knowledge use and reporting of ProFaNE COS domains in a purposeful sample of RCTs. Our aim was to investigate factors governing implementation fidelity. However, implementation fidelity represents only one of several important implementation outcomes. Different methodological approaches are needed to explore other outcomes of successful implementation, including adoption (i.e. uptake) or penetration (i.e. reach) of the ProFaNE COS. These outcomes may be more appropriately assessed via a review of clinical trial registry data to explore what domains are purportedly included in all trials registered after 2005 that fit the scope of the ProFaNE COS [48].

Our study has several limitations. This study was not pre-registered, so we are unable to document whether the study methods were not changed after the study began. The observational nature of our research design means we have provided evidence of association, not causation. We have assumed that citation is evidence of adoption (i.e. uptake), such that clinical trialists who cited the ProFaNE COS had made an initial decision to try to employ it in their trial design. However, it is possible that some trialists became aware of the ProFaNE COS too late for it to have influenced their trial design. Our approach also failed to identify trialists who implemented the ProFaNE COS in their trial design but chose not to cite it when disseminating their study results. We were restricted to investigating the potential role of factors easily abstractable from peer-reviewed academic articles, even though the CFIR states that determinants of knowledge use are vast and span diverse levels of influence [36]. We intend to build on the findings of this study by interviewing corresponding authors of included articles to better understand barriers and enablers to implementation fidelity of the ProFaNE COS. Our sample size was not large enough to dependably handle a complex statistical model containing all of our explanatory variables. To overcome this limitation, we employed commonly used variable selection methods which we acknowledge can be problematic in terms of overfitting and the inclusion of nuisance variables in the models [49, 50]. To minimize this risk, we used variable selection methods that are penalized (e.g. LASSO) and cross-checked our results using multiple techniques to select the most parsimonious models.

## Conclusion

By way of a systematic citation analysis of the ProFaNE COS and a correlational study, we found that amongst the subset of articles disseminating the results of RCTs in older people, the number (%) of ProFaNE COS domains reported ranged from 0 to 5 (0–100%), with a median of 2 (40%). We also observed that RCTs funded by at least one non-industry source, examining exercise or multi-component/factorial interventions, reported the highest mean percentages of ProFaNE COS domains. Findings may help inform strategies to increase the impact of the ProFaNE COS, which should lead to more consistent outcome reporting across RCTs and, ultimately, better understanding of the effectiveness and safety of interventions to prevent and/or manage falls in older people.

## Supplementary Information

**Additional file 1. **Modelfit characteristics for our model on the percentage of ProFaNE COS domainsreported.

**Additional file 2. **Modelfit characteristics for our models on likelihood of reporting each domain ofthe ProFaNE COS.

**Additional file 3. **Listof included articles (*n*=85).

**Additional file 4. **Listof articles excluded after full-text review (*n*=131).

## Data Availability

The datasets used and/or analysed during the current study are available from the corresponding author on reasonable request.

## Abbreviations

CFIR: Consolidated Framework for Implementation Research
CI: Confidence interval
COS: Core outcome set
COMET: Core Outcome Measures in Effectiveness Trials
HRQoL: Health-related quality of life
HTA: Health Technology Assessment
NIHR: National Institute for Health Research
OR: Odds ratio
PCORI: Patient-Centered Outcomes Research Institute
ProFaNE: Prevention of Falls Network Europe
RCT: Randomized controlled trial
SD: Standard deviation
UK: United Kingdom
US: United States
