# Supplementary material for: Factors associated with reporting of the Prevention of Falls Network Europe (ProFaNE) core outcome set domains in randomized trials on falls in older people: a citation analysis and correlational study
Source: Trials. 2022 Aug 26;23:710. doi: 10.1186/s13063-022-06642-w (PMC9419335; doi:10.1186/s13063-022-06642-w)

Appendix Figure 1: Best model fit criterion has been met for most of the criterion.

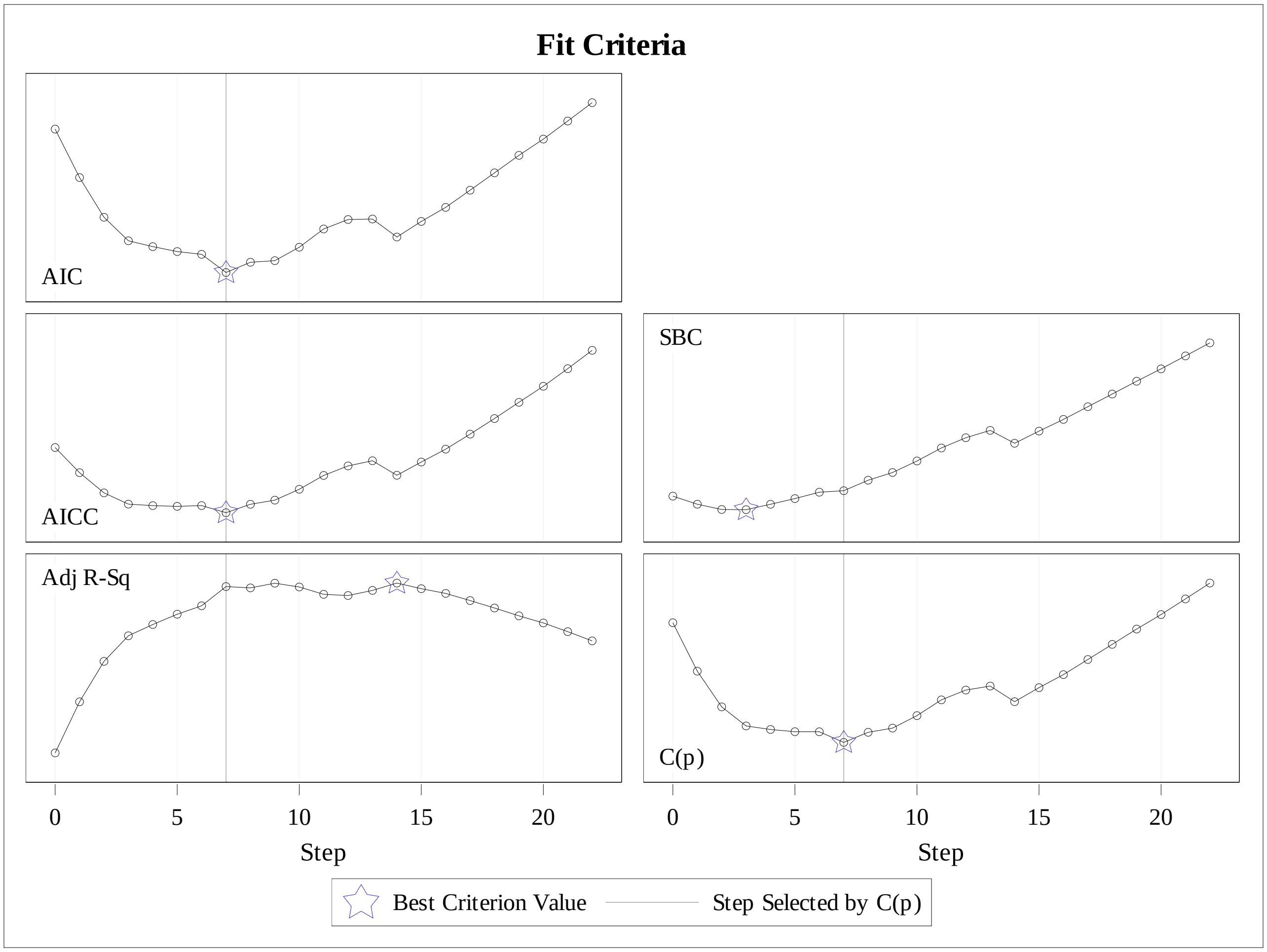

Appendix Figure 2: Average squared error was minimum at chosen step.

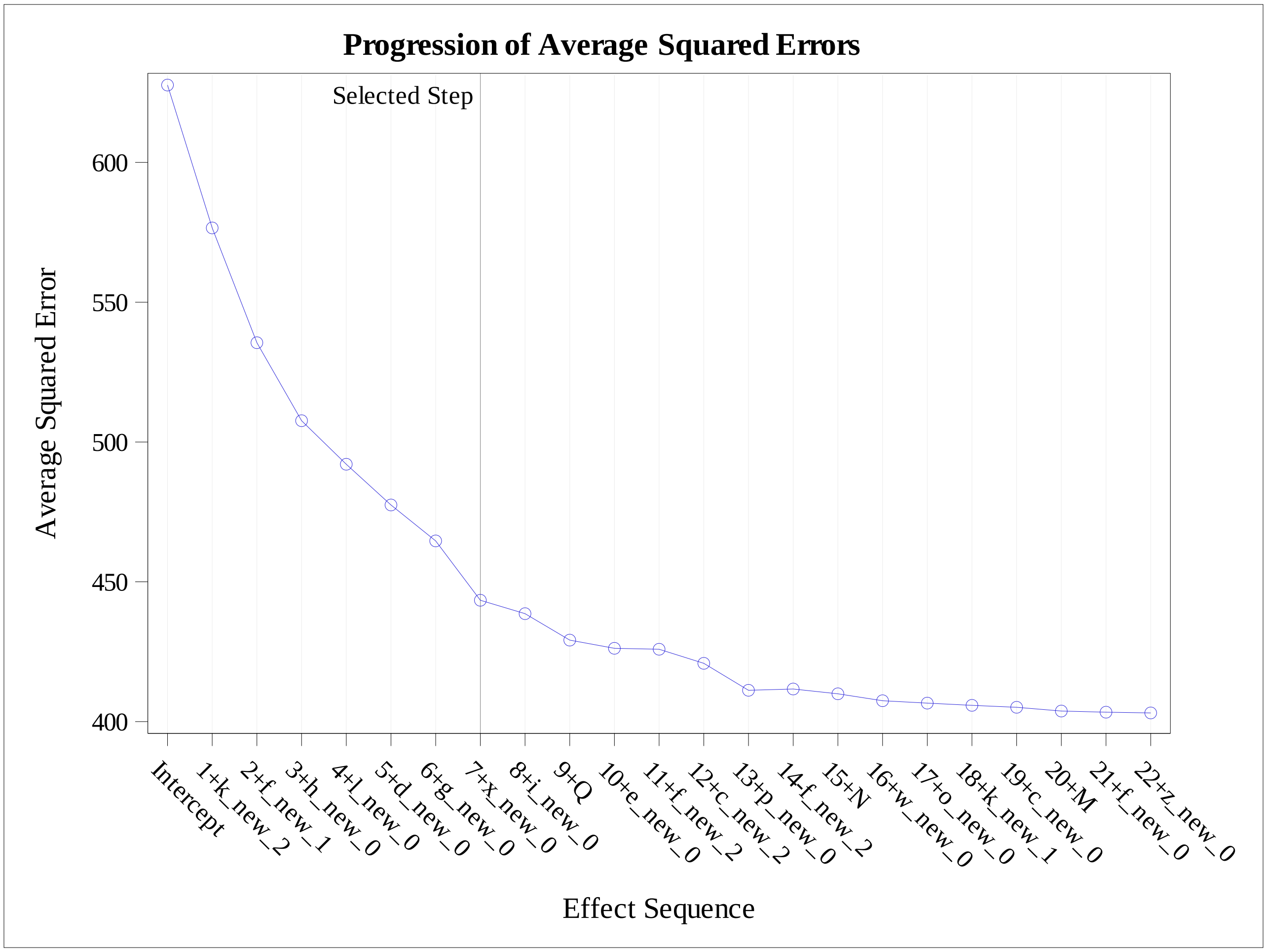

Supplement: Supplementary file 1 — Additional file 1. Modelfit characteristics for our model on the percentage of ProFaNE COS domainsreported. [file 13063_2022_6642_MOESM1_ESM.pdf]
