# Supplementary material for: Factors associated with reporting of the Prevention of Falls Network Europe (ProFaNE) core outcome set domains in randomized trials on falls in older people: a citation analysis and correlational study
Source: Trials. 2022 Aug 26;23:710. doi: 10.1186/s13063-022-06642-w (PMC9419335; doi:10.1186/s13063-022-06642-w)

Appendix figure 3: Fitted model with classification statistics for response variable Fall reported.

Fit Statistics									
	N Leaves	ASE	Mis-class	Sensitivity	Specificity	Entropy	Gini	RSS	AUC
**Model Based**	10	0.0149	0.0238	0.9733	1.0000	0.0624	0.0298	2.5000	0.9956
**Cross Validation**	10	0.1546	0.1522	0.9067	0.2222				

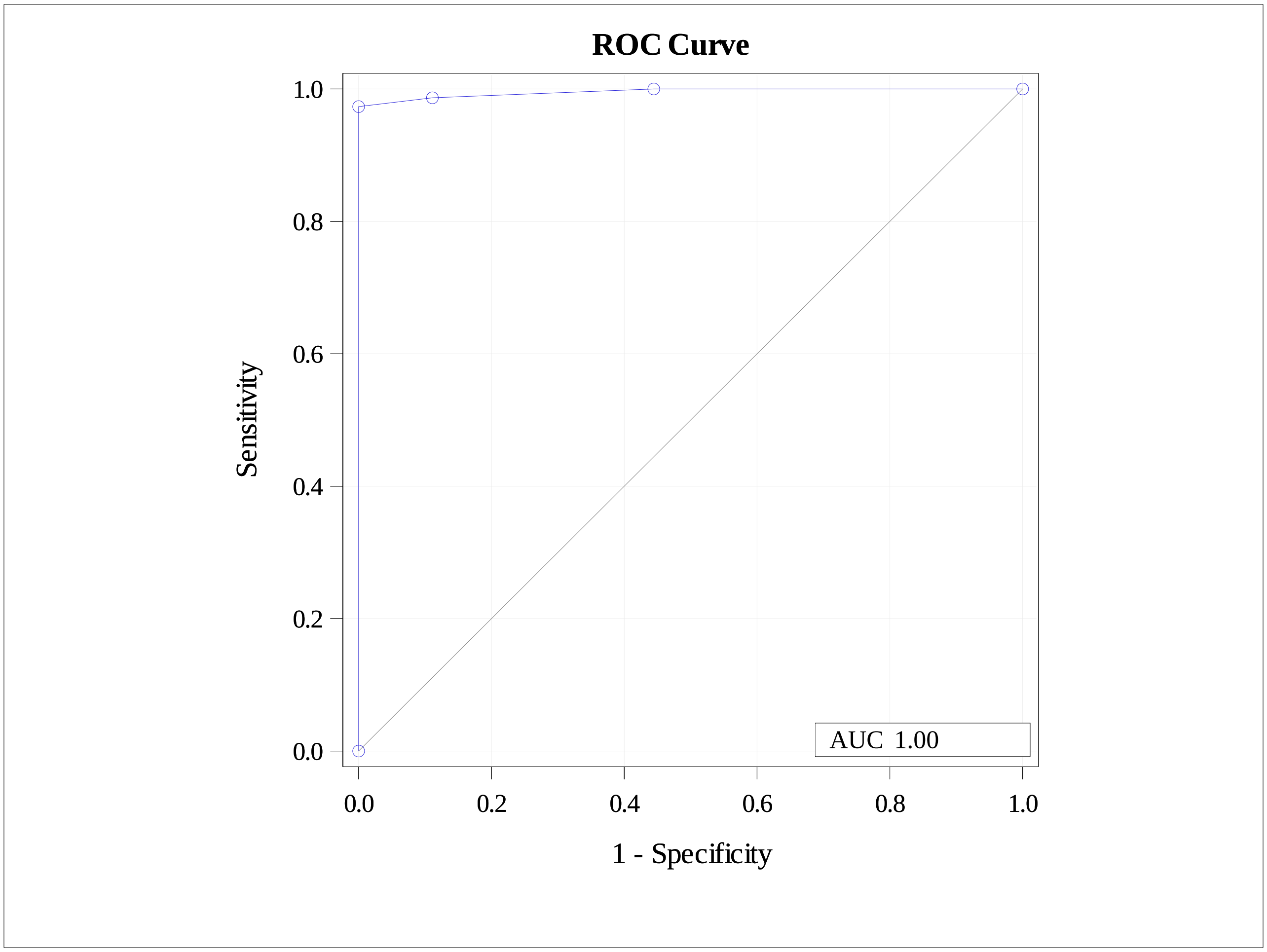

Appendixfigure 4: Fitted model with classification statistics for response variable Injuries reported.

Fit Statistics									
	N Leaves	ASE	Mis-class	Sensitivity	Specificity	Entropy	Gini	RSS	AUC
**Model Based**	10	0.0912	0.1429	0.8511	0.8649	0.4045	0.1824	15.3183	0.9416
**Cross Validation**	10	0.2694	0.3359	0.6596	0.7027				

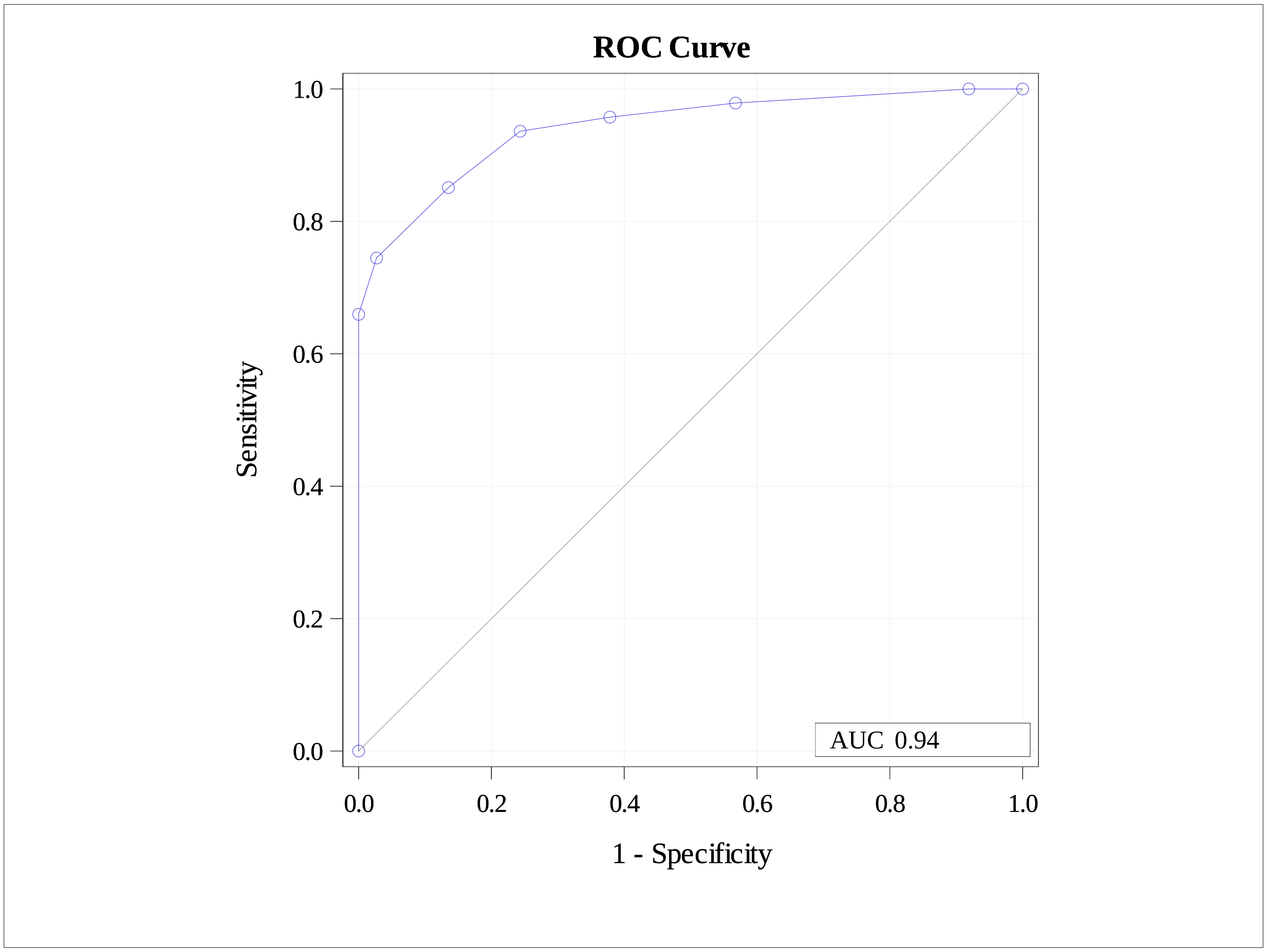

Appendixfigure 5: Fitted model with classification statistics for response variable Psychological Consequences of falling reported.

Fit Statistics									
	N Leaves	ASE	Mis-class	Sensitivity	Specificity	Entropy	Gini	RSS	AUC
**Model Based**	10	0.1223	0.1667	0.9211	0.7609	0.5403	0.2447	20.5516	0.8890
**Cross Validation**	10	0.3824	0.4675	0.4474	0.6304				

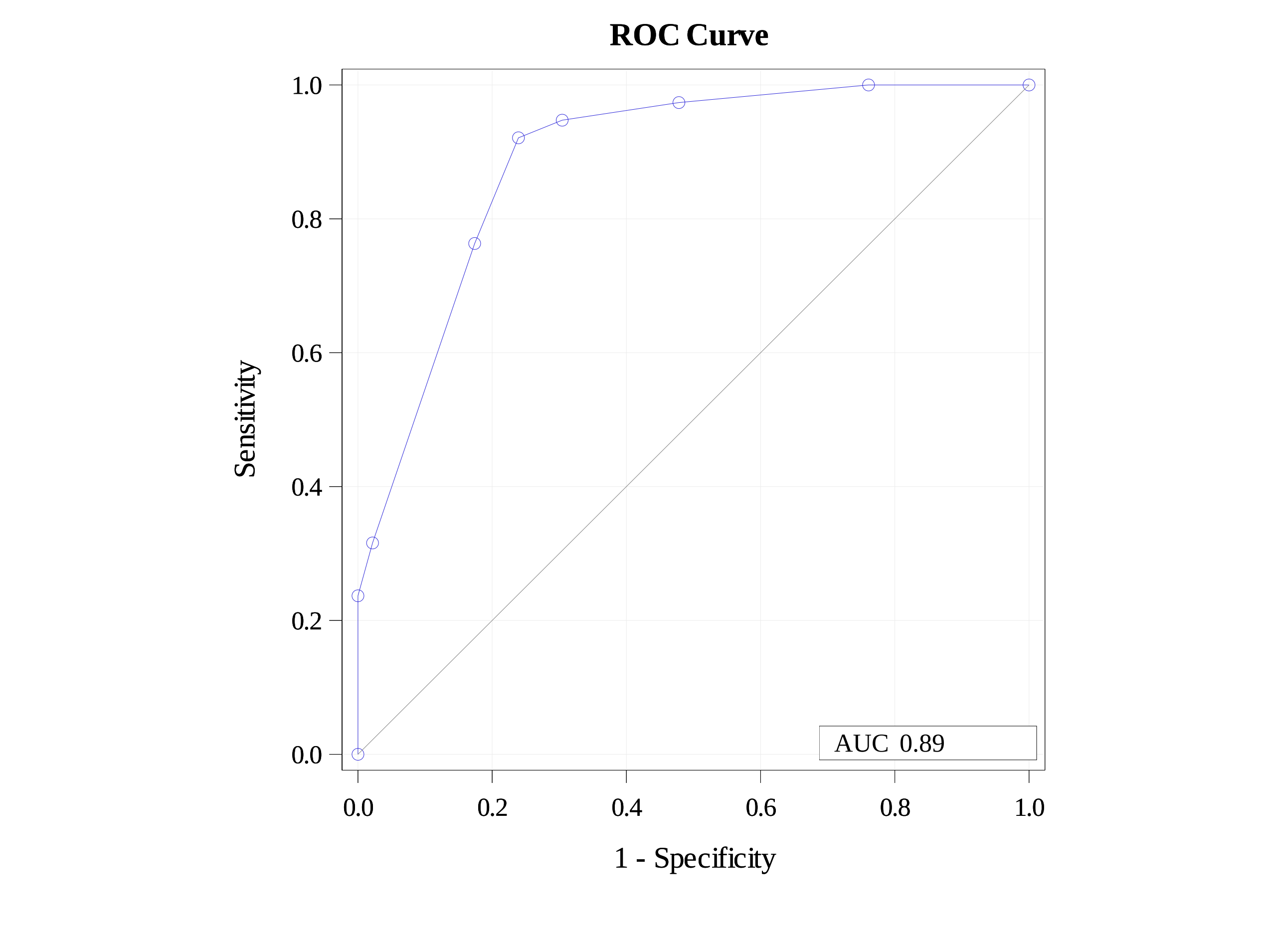

Appendix figure 6: Fitted model with classification statistics for response variable QOL reported.

Fit Statistics									
	N Leaves	ASE	Mis-class	Sensitivity	Specificity	Entropy	Gini	RSS	AUC
**Model Based**	10	0.1268	0.1667	0.5862	0.9636	0.5665	0.2536	21.2987	0.8533
**Cross Validation**	10	0.3540	0.4740	0.2759	0.6545				

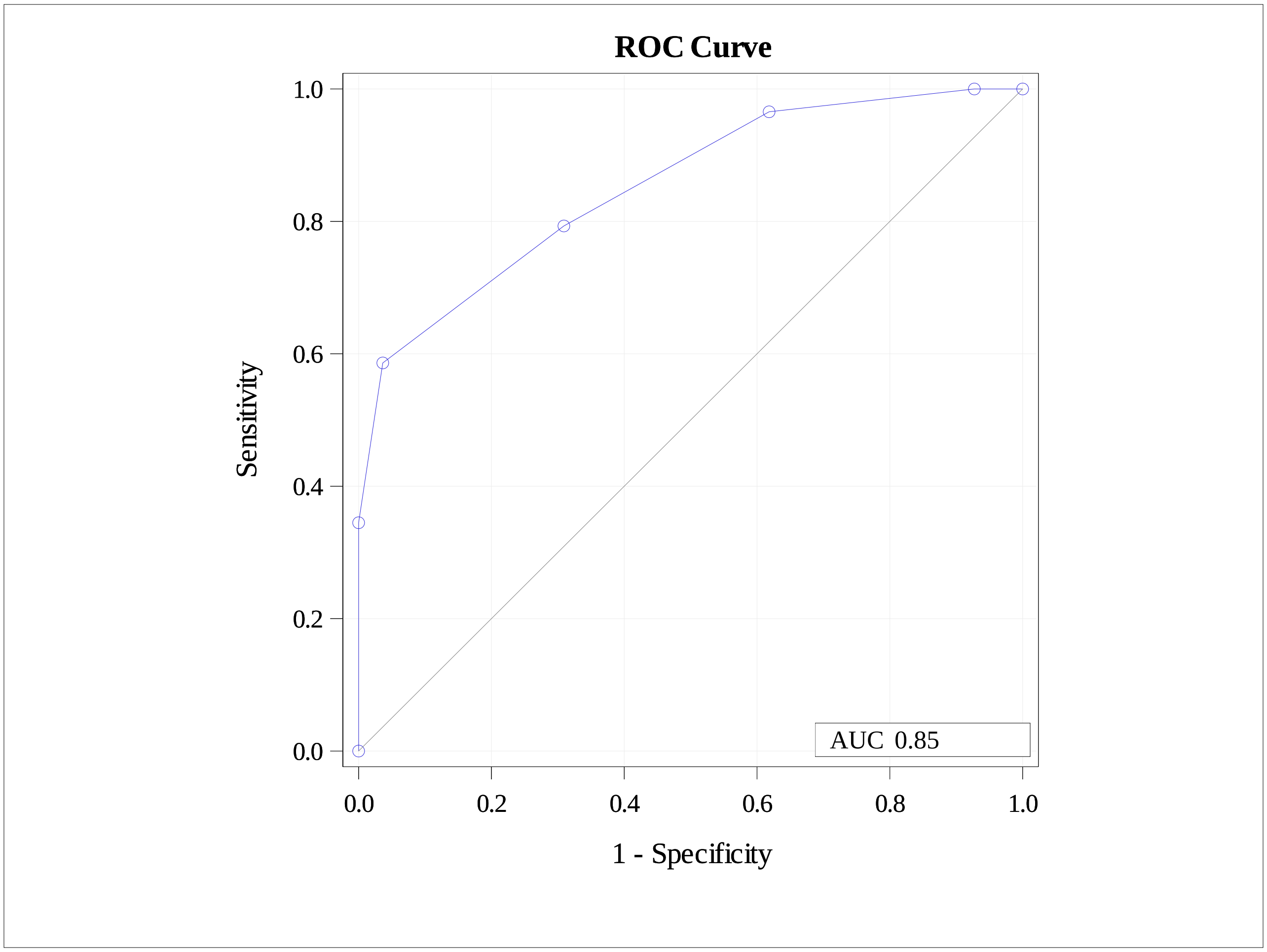

Appendix figure 7: Fitted model with classification statistics for response variable Physical Activity reported.

Fit Statistics									
	N Leaves	ASE	Mis-class	Sensitivity	Specificity	Entropy	Gini	RSS	AUC
**Model Based**	20	0	0.0000	1.0000	1.0000	0	0	0	1.0000
**Cross Validation**	19	0.4848	0.4867	0.4839	0.5283				

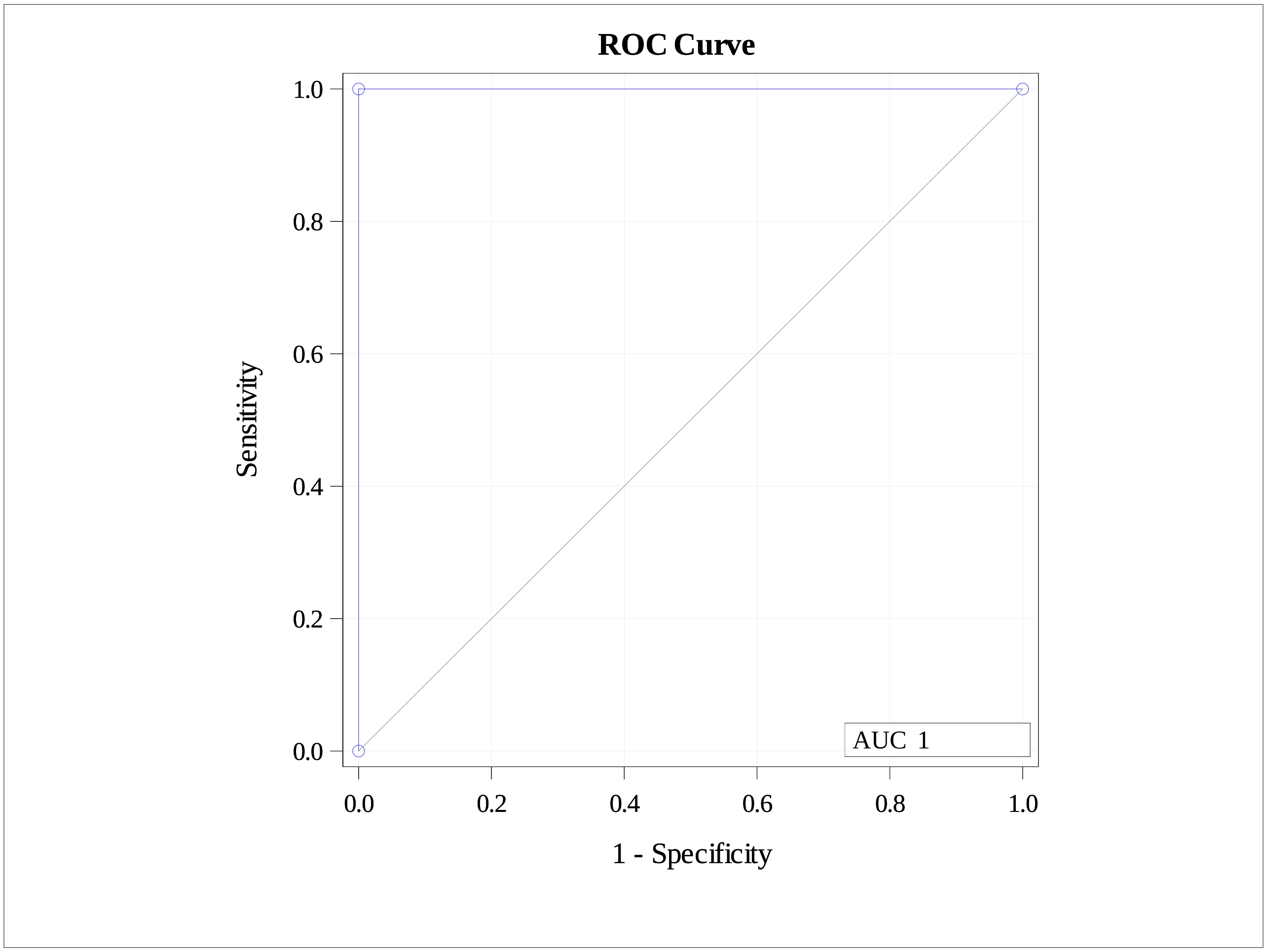

Supplement: Supplementary file 2 — Additional file 2. Modelfit characteristics for our models on likelihood of reporting each domain ofthe ProFaNE COS. [file 13063_2022_6642_MOESM2_ESM.pdf]
