# Supplementary material for: Factors associated with reporting of the Prevention of Falls Network Europe (ProFaNE) core outcome set domains in randomized trials on falls in older people: a citation analysis and correlational study
Source: Trials. 2022 Aug 26;23:710. doi: 10.1186/s13063-022-06642-w (PMC9419335; doi:10.1186/s13063-022-06642-w)
Supplement: Supplementary file 3 — Additional file 3. Listof included articles (n=85). [file 13063_2022_6642_MOESM3_ESM.pdf]

Additional file 1: List of included articles (n=85)

1. Arkkukangas M, Söderlund A, Eriksson S, Johansson AC. Fall preventive exercise with or without behavior change support for community-dwelling older adults: A randomized controlled trial with short-term follow-up. Journal of Geriatric Physical Therapy. 2019;42(1):9-17.
2. Barban F, Annicchiarico R, Melideo M, Federici A, Lombardi MG, Giuli S, et al. Reducing fall risk with combined motor and cognitive training in elderly fallers. Brain Sci. 2017;7(2).
3. Batchelor FA, Hill KD, Mackintosh SF, Said CM, Whitehead CH. Effects of a multifactorial falls prevention program for people with stroke returning home after rehabilitation: A randomized controlled trial. Arch Phys Med Rehabil. 2012;93(9):1648-55.
4. Bhasin S, Gill TM, Reuben DB, Latham NK, Ganz DA, Greene EJ, et al. A randomized trial of a multifactorial strategy to prevent serious fall injuries. N Engl J Med. 2020;383(2):129-40.
5. Cockayne S, Adamson J, Clarke A, Corbacho B, Fairhurst C, Green L, et al. Cohort randomised controlled trial of a multifaceted podiatry intervention for the prevention of falls in older people (The REFORM Trial). PLoS One. 2017;12(1):e0168712.
6. Cohen MA, Miller J, Shi X, Sandhu J, Lipsitz LA. Prevention program lowered the risk of falls and decreased claims for long-term services among elder participants. Health Aff (Millwood). 2015;34(6):971-7.
7. Condurache CI, Chiu S, Chotiyarnwong P, Johansson H, Shepstone L, Lenaghan E, et al. Screening for high hip fracture risk does not impact on falls risk: A post hoc analysis from the SCOOP study. Osteoporosis International. 2020;31(3):457-64.
8. Del Din S, Galna B, Lord S, Nieuwboer A, Bekkers EMJ, Pelosin E, et al. Falls risk in relation to activity exposure in high-risk older adults. Journals of Gerontology - Series A Biological Sciences and Medical Sciences. 2020;75(6):1198-205.

9. Delbaere K, Valenzuela T, Lord SR, Clemson L, Zijlstra GAR, Close JCT, et al. E-health StandingTall balance exercise for fall prevention in older people: Results of a two year randomised controlled trial. BMJ. 2021;373:n740.

10. Dorresteijn TA, Zijlstra GA, Ambergen AW, Delbaere K, Vlaeyen JW, Kempen GI. Effectiveness of a home-based cognitive behavioral program to manage concerns about falls in community-dwelling, frail older people: Results of a randomized controlled trial. BMC Geriatr. 2016;16:2.

11. Duque G, Boersma D, Loza-Diaz G, Hassan S, Suarez H, Geisinger D, et al. Effects of balance training using a virtual-reality system in older fallers. Clin Interv Aging. 2013;8:257-63.

12. El-Khoury F, Cassou B, Latouche A, Aegerter P, Charles MA, Dargent-Molina P. Effectiveness of two year balance training programme on prevention of fall induced injuries in at risk women aged 75-85 living in community: Ossebo randomised controlled trial. BMJ. 2015;351:h3830.

13. Elley CR, Robertson MC, Garrett S, Kerse NM, McKinlay E, Lawton B, et al. Effectiveness of a falls-and-fracture nurse coordinator to reduce falls: A randomized, controlled trial of at-risk older adults. J Am Geriatr Soc. 2008;56(8):1383-9.

14. Faes MC, Reelick MF, Melis RJ, Borm GF, Esselink RA, Rikkert MG. Multifactorial fall prevention for pairs of frail community-dwelling older fallers and their informal caregivers: A dead end for complex interventions in the frailest fallers. J Am Med Dir Assoc. 2011;12(6):451-8.

15. Ferrer A, Formiga F, Sanz H, de Vries OJ, Badia T, Pujol R, et al. Multifactorial assessment and targeted intervention to reduce falls among the oldest-old: A randomized controlled trial. Clin Interv Aging. 2014;9:383-93.

16. Fitzharris MP, Day L, Lord SR, Gordon I, Fildes B. The Whitehorse NoFalls trial: Effects on fall rates and injurious fall rates. Age Ageing. 2010;39(6):728-33.

17. Freiberger E, Haberle L, Spirduso WW, Zijlstra GA. Long-term effects of three multicomponent exercise interventions on physical performance and fall-related psychological outcomes in community-dwelling older adults: A randomized controlled trial. J Am Geriatr Soc. 2012;60(3):437-46.

18. Freiberger E, Menz HB, Abu-Omar K, Rutten A. Preventing falls in physically active community-dwelling older people: A comparison of two intervention techniques. Gerontology. 2007;53(5):298-305.

19. Gill TM, Pahor M, Guralnik JM, McDermott MM, King AC, Buford TW, et al. Effect of structured physical activity on prevention of serious fall injuries in adults aged 70-89: Randomized clinical trial (LIFE Study). BMJ. 2016;352:i245.

20. Goodwin VA, Richards SH, Henley W, Ewings P, Taylor AH, Campbell JL. An exercise intervention to prevent falls in people with Parkinson's disease: A pragmatic randomised controlled trial. J Neurol Neurosurg Psychiatry. 2011;82(11):1232-8.

21. Harper KJ, Arendts G, Geelhoed EA, Barton AD, Celenza A. Cost analysis of a brief intervention for the prevention of falls after discharge from an emergency department. Journal of Evaluation in Clinical Practice. 2019;25(2):244-50.

22. Harper KJ, Barton AD, Arendts G, Edwards DG, Petta AC, Celenza A. Controlled clinical trial exploring the impact of a brief intervention for prevention of falls in an emergency department. Emerg Med Australas. 2017;29(5):524-30.

23. Harper KJ, Barton AD, Bharat C, Petta AC, Edwards DG, Arendts G, et al. Risk assessment and the impact of point of contact intervention following emergency department presentation with a fall. Physical & Occupational Therapy in Geriatrics. 2017;35(3-4):182-94.

24. Hewitt J, Goodall S, Clemson L, Henwood T, Refshauge K. Progressive resistance and balance training for falls prevention in long-term residential aged care: A cluster randomized trial of the Sunbeam program. J Am Med Dir Assoc. 2018;19(4):361-9.

25. Hill AM, Hoffmann T, McPhail S, Beer C, Hill KD, Oliver D, et al. Evaluation of the sustained effect of inpatient falls prevention education and predictors of falls after hospital discharge--Follow-up to a randomized controlled trial. J Gerontol A Biol Sci Med Sci. 2011;66(9):1001-12.

26. Hill AM, McPhail SM, Haines TP, Morris ME, Etherton-Beer C, Shorr R, et al. Falls after hospital discharge: A randomized clinical trial of individualized multimodal falls prevention education. Journals of Gerontology - Series A Biological Sciences and Medical Sciences. 2019;74(9):1511-7.

27. Hinrichs T, Bucker B, Klaassen-Mielke R, Brach M, Wilm S, Platen P, et al. Home-based exercise supported by general practitioner practices: Ineffective in a sample of chronically ill, mobility-limited older adults (the HOMEfit randomized controlled trial). J Am Geriatr Soc. 2016;64(11):2270-9.

28. Hwang HF, Chen SJ, Lee-Hsieh J, Chien DK, Chen CY, Lin MR. Effects of home-based tai chi and lower extremity training and self-practice on falls and functional outcomes in older fallers from the emergency department-A randomized controlled trial. J Am Geriatr Soc. 2016;64(3):518-25.

29. Josephs S, Pratt ML, Calk Meadows E, Thurmond S, Wagner A. The effectiveness of Pilates on balance and falls in community dwelling older adults. J Bodyw Mov Ther. 2016;20(4):815-23.

30. Kapan A, Luger E, Haider S, Titze S, Schindler K, Lackinger C, et al. Fear of falling reduced by a lay led home-based program in frail community-dwelling older adults: A randomised controlled trial. Arch Gerontol Geriatr. 2017;68:25-32.

31. Kemmler W, von Stengel S, Engelke K, Haberle L, Kalender WA. Exercise effects on bone mineral density, falls, coronary risk factors, and health care costs in older women: The randomized controlled Senior Fitness and Prevention (SEFIP) study. Arch Intern Med. 2010;170(2):179-85.

32. Kovács E, Prókai L, Mészáros L, Gondos T. Adapted physical activity is beneficial on balance, functional mobility, quality of life and fall risk in community-dwelling older women: A randomized single-blinded controlled trial. Eur J Phys Rehabil Med. 2013;49(3):301-10.

33. Kovács E, Sztruhár Jónásné I, Karóczi CK, Korpos A, Gondos T. Effects of a multimodal exercise program on balance, functional mobility and fall risk in older adults with cognitive impairment: A randomized controlled single-blind study. Eur J Phys Rehabil Med. 2013;49(5):639-48.

34. Lamb SE, Bruce J, Hossain A, Ji C, Longo R, Lall R, et al. Screening and intervention to prevent falls and fractures in older people. New England Journal of Medicine. 2020;383(19):1848-59.

35. Lockwood KJ, Harding KE, Boyd JN, Taylor NF. Predischarge home visits after hip fracture: A randomized controlled trial. Clinical Rehabilitation. 2019;33(4):681-92.

36. Logan PA, Coupland CA, Gladman JR, Sahota O, Stoner-Hobbs V, Robertson K, et al. Community falls prevention for people who call an emergency ambulance after a fall: Randomised controlled trial. BMJ. 2010;340:c2102.

37. Lurie JD, Zagaria AB, Ellis L, Pidgeon D, Gill-Body KM, Burke C, et al. Surface perturbation training to prevent falls in older adults: A highly pragmatic, randomized controlled trial. Phys Ther. 2020;100(7):1153-62.

38. Luukinen H, Lehtola S, Jokelainen J, Vaananen-Sainio R, Lotvonen S, Koistinen P. Pragmatic exercise-oriented prevention of falls among the elderly: A population-based, randomized, controlled trial. Prev Med. 2007;44(3):265-71.

39. Mackey DC, Lachance CC, Wang PWT, Feldman F, Laing AC, Leung PM, et al. The Flooring for Injury Prevention (FLIP) Study of compliant flooring for the prevention of fall-related injuries in long-term care: A randomized trial. Plos Medicine. 2019;16(6):20.

40. Mansfield A, Aqui A, Danells CJ, Knorr S, Centen A, DePaul VG, et al. Does perturbation-based balance training prevent falls among individuals with chronic stroke? A randomised controlled trial. BMJ Open. 2018;8(8):e021510.

41. McMurdo ME, Price RJ, Shields M, Potter J, Stott DJ. Should oral nutritional supplementation be given to undernourished older people upon hospital discharge? A controlled trial. J Am Geriatr Soc. 2009;57(12):2239-45.

42. Merom D, Mathieu E, Cerin E, Morton RL, Simpson JM, Rissel C, et al. Social dancing and incidence of falls in older adults: A cluster randomised controlled trial. PLoS Med. 2016;13(8):e1002112.

43. Mikolaizak AS, Lord SR, Tiedemann A, Simpson P, Caplan GA, Bendall J, et al. A multidisciplinary intervention to prevent subsequent falls and health service use following fall-related paramedic care: A randomised controlled trial. Age Ageing. 2017;46(2):200-7.

44. Moller UO, Kristensson J, Midlov P, Ekdahl C, Jakobsson U. Effects of a one-year home-based case management intervention on falls in older people: A randomized controlled trial. J Aging Phys Act. 2014;22(4):457-64.

45. Nyman SR, Ingram W, Sanders J, Thomas PW, Thomas S, Vassallo M, et al. Randomised controlled trial of the effect of Tai Chi on postural balance of people with dementia. Clin Interv Aging. 2019;14:2017-29.

46. Oliveira JS, Sherrington C, Paul SS, Ramsay E, Chamberlain K, Kirkham C, et al. A combined physical activity and fall prevention intervention improved mobility-related goal attainment but not physical activity in older adults: A randomised trial. Journal of Physiotherapy. 2019;65(1):16-22.

47. Palvanen M, Kannus P, Piirtola M, Niemi S, Parkkari J, Jarvinen M. Effectiveness of the Chaos Falls Clinic in preventing falls and injuries of home-dwelling older adults: A randomised controlled trial. Injury. 2014;45(1):265-71.

48. Parry SW, Bamford C, Deary V, Finch TL, Gray J, MacDonald C, et al. Cognitive-behavioural therapy-based intervention to reduce fear of falling in older people: Therapy development and randomised controlled trial - the Strategies for Increasing Independence, Confidence and Energy (STRIDE) study. Health Technol Assess. 2016;20(56):1-206.

49. Parsons J, Mathieson S, Jull A, Parsons M. Does vibration training reduce the fall risk profile of frail older people admitted to a rehabilitation facility? A randomised controlled trial. Disabil Rehabil. 2016;38(11):1082-8.

50. Patil R, Uusi-Rasi K, Tokola K, Karinkanta S, Kannus P, Sievanen H. Effects of a multimodal exercise program on physical function, falls, and injuries in older women: A 2-year community-based, randomized controlled trial. J Am Geriatr Soc. 2015;63(7):1306-13.

51. Perula LA, Varas-Fabra F, Rodriguez V, Ruiz-Moral R, Fernandez JA, Gonzalez J, et al. Effectiveness of a multifactorial intervention program to reduce falls incidence among community-living older adults: A randomized controlled trial. Arch Phys Med Rehabil. 2012;93(10):1677-84.

52. Polinder S, Boye ND, Mattace-Raso FU, Van der Velde N, Hartholt KA, De Vries OJ, et al. Cost-utility of medication withdrawal in older fallers: Results from the improving medication prescribing to reduce risk of FALLs (IMPROveFALL) trial. BMC Geriatr. 2016;16(1):179.

53. Rapp K, Lamb SE, Buchele G, Lall R, Lindemann U, Becker C. Prevention of falls in nursing homes: Subgroup analyses of a randomized fall prevention trial. J Am Geriatr Soc. 2008;56(6):1092-7.

54. Schepens SL, Panzer V, Goldberg A. Randomized controlled trial comparing tailoring methods of multimedia-based fall prevention education for community-dwelling older adults. Am J Occup Ther. 2011;65(6):702-9.

55. Scragg R. The Vitamin D Assessment (ViDA) study - Design and main findings. Journal of Steroid Biochemistry and Molecular Biology. 2020;198:6.

56. Shigematsu R, Okura T, Nakagaichi M, Tanaka K, Sakai T, Kitazumi S, et al. Square-stepping exercise and fall risk factors in older adults: A single-blind, randomized controlled trial. J Gerontol A Biol Sci Med Sci. 2008;63(1):76-82.

57. Shigematsu R, Okura T, Sakai T, Rantanen T. Square-stepping exercise versus strength and balance training for fall risk factors. Aging Clin Exp Res. 2008;20(1):19-24.

58. Siegrist M, Freiberger E, Geilhof B, Salb J, Hentschke C, Landendoerfer P, et al. Fall prevention in a primary care setting. Dtsch Arztebl Int. 2016;113(21):365-72.

59. Sitja-Rabert M, Martinez-Zapata MJ, Fort Vanmeerhaeghe A, Rey Abella F, Romero-Rodriguez D, Bonfill X. Effects of a whole body vibration (WBV) exercise intervention for institutionalized older people: A randomized, multicentre, parallel, clinical trial. J Am Med Dir Assoc. 2015;16(2):125-31.

60. Smulders E, Weerdesteyn V, Groen BE, Duysens J, Eijsbouts A, Laan R, et al. Efficacy of a short multidisciplinary falls prevention program for elderly persons with osteoporosis and a fall history: A randomized controlled trial. Arch Phys Med Rehabil. 2010;91(11):1705-11.

61. Snooks HA, Anthony R, Chatters R, Dale J, Fothergill R, Gaze S, et al. Support and Assessment for Fall Emergency Referrals (SAFER) 2: A cluster randomised trial and systematic review of clinical effectiveness and cost-effectiveness of new protocols for emergency ambulance paramedics to assess older people following a fall with referral to community-based care when appropriate. Health Technol Assess. 2017;21(13):1-218.

62. Sousa N, Mendes R, Silva A, Oliveira J. Combined exercise is more effective than aerobic exercise in the improvement of fall risk factors: A randomized controlled trial in community-dwelling older men. Clin Rehabil. 2017;31(4):478-86.

63. Sparrow D, DeAngelis TR, Hendron K, Thomas CA, Saint-Hilaire M, Ellis T. Highly challenging balance program reduces fall rate in Parkinson Disease. J Neurol Phys Ther. 2016;40(1):24-30.

64. Spink MJ, Menz HB, Fotoohabadi MR, Wee E, Landorf KB, Hill KD, et al. Effectiveness of a multifaceted podiatry intervention to prevent falls in community dwelling older people with disabling foot pain: Randomised controlled trial. BMJ. 2011;342:d3411.

65. Stanmore EK, Mavroeidi A, de Jong LD, Skelton DA, Sutton CJ, Benedetto V, et al. The effectiveness and cost-effectiveness of strength and balance Exergames to reduce falls risk for

people aged 55 years and older in UK assisted living facilities: A multi-centre, cluster randomised controlled trial. BMC Med. 2019;17(1):49.

66. Sumukadas D, Price R, McMurdo MET, Rauchhaus P, Struthers A, McSwiggan S, et al. The effect of perindopril on postural instability in older people with a history of falls-A randomised controlled trial. Age Ageing. 2018;47(1):75-81.

67. Taylor D, Hale L, Schluter P, Waters DL, Binns EE, McCracken H, et al. Effectiveness of Tai Chi as a community-based falls prevention intervention: A randomized controlled trial. J Am Geriatr Soc. 2012;60(5):841-8.

68. Taylor ME, Wesson J, Sherrington C, Hill KD, Kurrle S, Lord SR, et al. Tailored exercise and home hazard reduction program for fall prevention in older people with cognitive impairment: The i-FOCIS randomized controlled trial. Journals of Gerontology - Series A Biological Sciences and Medical Sciences. 2021;76(4):655-65.

69. Tilson JK, Wu SS, Cen SY, Feng Q, Rose DR, Behrman AL, et al. Characterizing and identifying risk for falls in the LEAPS study: A randomized clinical trial of interventions to improve walking post stroke. Stroke. 2012;43(2):446-52.

70. Toots A, Wiklund R, Littbrand H, Nordin E, Nordström P, Lundin-Olsson L, et al. The effects of exercise on falls in older people with dementia living in nursing homes: A randomized controlled trial. Journal of the American Medical Directors Association. 2019;20(7):835-42.e1.

71. Trombetti A, Hars M, Herrmann FR, Kressig RW, Ferrari S, Rizzoli R. Effect of music-based multitask training on gait, balance, and fall risk in elderly people: A randomized controlled trial. Arch Intern Med. 2011;171(6):525-33.

72. Tuvemo Johnson S, Anens E, Johansson AC, Hellström K. The Otago exercise program with or without motivational interviewing for community-dwelling older adults: A 12-month follow-up of a randomized, controlled trial. J Appl Gerontol. 2021;40(3):289-99.

73. Uusi-Rasi K, Patil R, Karinkanta S, Kannus P, Tokola K, Lamberg-Allardt C, et al. Exercise and vitamin D in fall prevention among older women: A randomized clinical trial. JAMA Intern Med. 2015;175(5):703-11.

74. Uusi-Rasi K, Patil R, Karinkanta S, Kannus P, Tokola K, Lamberg-Allardt C, et al. A 2-year follow-up after a 2-year RCT with vitamin D and exercise: Effects on falls, injurious falls and physical functioning among older women. J Gerontol A Biol Sci Med Sci. 2017;72(9):1239-45.

75. Vaapio S, Salminen M, Vahlberg T, Sjosten N, Isoaho R, Aarnio P, et al. Effects of risk-based multifactorial fall prevention on health-related quality of life among the community-dwelling aged: A randomized controlled trial. Health Qual Life Outcomes. 2007;5:20.

76. van het Reve E, de Bruin ED. Strength-balance supplemented with computerized cognitive training to improve dual task gait and divided attention in older adults: A multicenter randomized-controlled trial. BMC Geriatr. 2014;14:134.

77. Verrusio W, Gianturco V, Cacciafesta M, Marigliano V, Troisi G, Ripani M. Fall prevention in the young old using an exoskeleton human body posturizer: A randomized controlled trial. Aging Clin Exp Res. 2017;29(2):207-14.

78. Vind AB, Andersen HE, Pedersen KD, Joergensen T, Schwarz P. Effect of a program of multifactorial fall prevention on health-related quality of life, functional ability, fear of falling and psychological well-being. A randomized controlled trial. Aging Clin Exp Res. 2010;22(3):249-54.

79. Vind AB, Andersen HE, Pedersen KD, Jorgensen T, Schwarz P. An outpatient multifactorial falls prevention intervention does not reduce falls in high-risk elderly Danes. J Am Geriatr Soc. 2009;57(6):971-7.

80. von Stengel S, Kemmler W, Engelke K, Kalender WA. Effects of whole body vibration on bone mineral density and falls: Results of the randomized controlled ELVIS study with postmenopausal women. Osteoporos Int. 2011;22(1):317-25.

81. Voukelatos A, Cumming RG, Lord SR, Rissel C. A randomized, controlled trial of Tai Chi for the prevention of falls: The Central Sydney Tai Chi trial. J Am Geriatr Soc. 2007;55(8):1185-91.

82. Voukelatos A, Merom D, Sherrington C, Rissel C, Cumming RG, Lord SR. The impact of a home-based walking programme on falls in older people: The Easy Steps randomised controlled trial. Age Ageing. 2015;44(3):377-83.

83. Wang C, Goel R, Zhang Q, Lepow B, Najafi B. Daily use of bilateral custom-made ankle-foot orthoses for fall prevention in older adults: A randomized controlled trial. J Am Geriatr Soc. 2019;67(8):1656-61.

84. Zieschang T, Schwenk M, Becker C, Uhlmann L, Oster P, Hauer K. Falls and physical activity in persons with mild to moderate dementia participating in an intensive motor training: Randomized controlled trial. Alzheimer Dis Assoc Disord. 2017;31(4):307-14.

85. Zieschang T, Schwenk M, Oster P, Hauer K. Sustainability of motor training effects in older people with dementia. J Alzheimers Dis. 2013;34(1):191-202.
