# Supplementary material for: Factors associated with reporting of the Prevention of Falls Network Europe (ProFaNE) core outcome set domains in randomized trials on falls in older people: a citation analysis and correlational study
Source: Trials. 2022 Aug 26;23:710. doi: 10.1186/s13063-022-06642-w (PMC9419335; doi:10.1186/s13063-022-06642-w)
Supplement: Supplementary file 4 — Additional file 4. Listof articles excluded after full-text review (n=131). [file 13063_2022_6642_MOESM4_ESM.pdf]

Additional file 2: List of articles excluded after full-text screening (n=131)

Articles excluded because not published in English (n=13)

1. Ardic T. Recurrent falls in the elderly person. Aide Soignante. 2018;32(198):25-7.
2. Becker C. [Prevention of falls and fall-related injuries: Personal balance and future tasks]. Z Gerontol Geriatr. 2017;50(8):672-5.
3. Dargent-Molina P, Cassou B. Prevention of falls and broken bones in older women: Ossebo random trials. Gerontologie et Societe. 2008;125:65-78.
4. Freiberger E, Sieber CC, Singler K. Mobility and Fall Prevention in older Community-dwelling Persons. Deutsche Medizinische Wochenschrift. 2020;145(13):932-43.
5. Heppner HJ. How fall-prone is your patient? MMW-Fortschritte der Medizin. 2018;160(11):19-20.
6. Kemmler W, S VS, Mayer S, Niedermayer M, Hentschke C, Kalender WA. [Effect of whole body vibration on the neuromuscular performance of females 65 years and older. One-year results of the controlled randomized ELVIS study]. Z Gerontol Geriatr. 2010;43(2):125-32.
7. Kemmler W, Stengel S, Mayer S, Engelke K, Kalender WA. Exercise effects on risk factors and health care costs in the elderly. Final results of the Senior Fitness and Prevention Study (SEFIP). Deutsche Zeitschrift fur Sportmedizin. 2010;61:264-9.
8. Kemmler W, von Stengel S. Validation of evidence-based recommendations in the setting of rehabilitation sports for people with osteoporosis: The randomised, controlled Senior Fitness and Prevention Study Senioren Fitness- und Präventions-Studie (SEFIP). Osteologie. 2020;29(03):215-20.

9. Perez-Ros P, Martinez-Arnau F, Tormos Minana I, Lopez Aracil A, Oltra Sanchis MC, Pechene Mera LE, et al. [Preliminary results of a community fall prevention programme: PRECAN study (falls prevention in La Ribera)]. Rev Esp Geriatr Gerontol. 2014;49(4):179-83.

10. Salva A, Rojano X, Coll-Planas L, Domenech S, Roque IFM. [Randomized clinical trial of a fall-prevention strategy for institutionalized elderly based on the Mini Falls Assessment Instrument]. Rev Esp Geriatr Gerontol. 2016;51(1):18-24.

11. Schone D, Freiberger E, Sieber CC. [Influence of skeletal muscles on the risk of falling in old age]. Internist (Berl). 2017;58(4):359-70.

12. Thiel C, Braun T, Grüneberg C. Physical training as core component of multimodal treatment of older frail people—study protocol of a randomized controlled pilot study. Zeitschrift fur Gerontologie und Geriatrie. 2019;52(1):45-60.

13. von Stengel S, Kemmler W, Mayer S, Engelke K, Klarner A, Kalender WA. [Effect of whole body vibration exercise on osteoporotic risk factors]. Dtsch Med Wochenschr. 2009;134(30):1511-6.

Articles excluded because not a randomized controlled trial (RCT) (n=27)

1. Avin KG, Hanke TA, Kirk-Sanchez N, McDonough CM, Shubert TE, Hardage J, et al. Management of falls in community-dwelling older adults: Clinical guidance statement from the Academy of Geriatric Physical Therapy of the American Physical Therapy Association. Phys Ther. 2015;95(6):815-34.

2. Campani D, Caristia S, Amariglio A, Piscone S, Ferrara LI, Barisone M, et al. Home and environmental hazards modification for fall prevention among the elderly. Public Health Nurs. 2021;38(3):493-501.

3. Carlsson G, Nilsson MH, Ekstam L, Chiatti C, Malmgren Fange A. Falls and fear of falling among persons who receive housing adaptations-Results from a quasi-experimental study in Sweden. Healthcare (Basel). 2017;5(4).

4. Carpenter CR, Cameron A, Ganz DA, Liu S. Older adult falls in emergency medicine—A sentinel event. Clinics in Geriatric Medicine. 2018;34(3):355-67.

5. Carpenter CR, Cameron A, Ganz DA, Liu S. Older adult falls in emergency medicine: 2019 update. Clinics in Geriatric Medicine. 2019;35(2):205-19.

6. Chen TY, Edwards JD, Janke MC. The effects of the A Matter of Balance program on falls and physical risk of falls, Tampa, Florida, 2013. Prev Chronic Dis. 2015;12:E157.

7. Eckert T, Kampe K, Kohler M, Albrecht D, Büchele G, Hauer K, et al. Correlates of fear of falling and falls efficacy in geriatric patients recovering from hip/pelvic fracture. Clinical Rehabilitation. 2020;34(3):416-25.

8. Hamm J, Money AG, Atwal A, Paraskevopoulos I. Fall prevention intervention technologies: A conceptual framework and survey of the state of the art. J Biomed Inform. 2016;59:319-45.

9. Hammarlund CS, Hagell P, Westergren A. Fall risk and its associated factors among older adults without home-help services in a Swedish municipality. J Community Health Nurs. 2016;33(4):181-9.

10. Hita-Contreras F, Martinez-Amat A, Cruz-Diaz D, Perez-Lopez FR. Fall prevention in postmenopausal women: The role of Pilates exercise training. Climacteric. 2016;19(3):229-33.

11. Huang MH. Balance and falls in survivors of cancer. Rehabilitation Oncology. 2021;39(1):4-5.

12. Isyar M, Saral I, Guler O, Cakar E, Mahirogullari M. Can prosthesis design of total knee arthroplasty affect balance? Eklem Hastalik Cerrahisi. 2015;26(2):72-6.

13. Lamb SE, Keene DJ. Measuring physical capacity and performance in older people. Best Pract Res Clin Rheumatol. 2017;31(2):243-54.

14. Lee A, Hayter M. Evaluating falls prevention strategies in community settings: Marginal reduction on rate of falls with individual risk-based multifactorial interventions compared to ‘usual care’. Evidence-Based Nursing. 2019;22(1):20.

15. Liu S, Yu X, Wang X, Li J, Jiang S, Kang L, et al. Intrinsic Capacity predicts adverse outcomes using Integrated Care for Older People screening tool in a senior community in Beijing. Archives of Gerontology and Geriatrics. 2021;94.

16. Lord S, Galna B, Yarnall AJ, Coleman S, Burn D, Rochester L. Have we been overestimating fall rates in Parkinson's disease? Mov Disord. 2017;32(5):803-4.

17. Martini D, Guareschi C, Biasini B, Bedogni G, Galli C, Angelino D, et al. Claimed effects, outcome variables and methods of measurement for health claims proposed under Regulation (EC) 1924/2006 in the framework of bone health. PharmaNutrition. 2018;6(1):17-36.

18. Mikolaizak AS, Lord SR, Tiedemann A, Simpson P, Caplan G, Bendall JC, et al. Adherence to a multifactorial fall prevention program following paramedic care: Predictors and impact on falls and health service use. Results from an RCT a priori subgroup analysis. Australasian Journal on Ageing. 2018;37(1):54-61.

19. Milius T, Mullendore C, Lazzarini I, Goodman C, Jensen L, Okada SH, et al. Considerations of Mobility. Occupational Therapy with Elders: Strategies for the COTA: Fourth Edition 2019. p. 186-216.

20. Nagaraj G, Hullick C, Arendts G, Burkett E, Hill KD, Carpenter CR. Avoiding anchoring bias by moving beyond ‘mechanical falls’ in geriatric emergency medicine. EMA - Emergency Medicine Australasia. 2018;30(6):843-50.

21. Ogilvie M, Wallen MP, Talpey SW. Agile ageing – A modifiable vital sign to mitigate the risk of falls in older adults? Medical Hypotheses. 2021;148.

22. Rome K, Morpeth T, Frecklington M. FRI0612-HPR Fear of falling and foot pain, impairment and disability in people with Rheumatoid Arthritis: An exploratory study. Annals of the Rheumatic Diseases. 2015;74(Suppl 2):1323.

23. Rudi T, Schaefer M, Manger B, Zink A, Strangfeld A. No increased risk of falls in patients treated with biologics compared to those under csDMARDs. Ann Rheum Dis. 2020;79:974.

24. Sherrington C, Tiedemann A, Fairhall NJ, Hopewell S, Michaleff ZA, Howard K, et al. Exercise for preventing falls in older people living in the community. Cochrane Database of Systematic Reviews. 2016(11).

25. Smith MD, Ben-Shlomo Y, Henderson E. How often are patients with progressive supranuclear palsy really falling? Journal of Neurology. 2019;266(8):2073-4.

26. Uemura K, Hasegawa T, Tougou H, Shuhei T, Uchiyama Y. Analysis of choice stepping with visual interference can detect prolonged postural preparation in older adults with mild cognitive impairment at high risk of falling. Dement Geriatr Cogn Disord. 2015;40(1-2):13-21.

27. Virág A, Karóczi CK, Jakab Á, Vass Z, Kovács É, Gondos T. Short-term and long-term effects of Nordic walking training on balance, functional mobility, muscle strength and aerobic endurance among Hungarian community-living older people: A feasibility study. J Sports Med Phys Fitness. 2015;55(11):1285-92.

Articles excluded because not older adult patient population (n=24)

1. Beauchamp MK, Brooks D, Ellerton C, Lee A, Alison J, Camp PG, et al. Pulmonary rehabilitation with balance training for fall reduction in chronic obstructive pulmonary disease: Protocol for a randomized controlled trial. JMIR Res Protoc. 2017;6(11):e228.

2. Carling A, Forsberg A, Gunnarsson M, Nilsagard Y. CoDuSe group exercise programme improves balance and reduces falls in people with Multiple Sclerosis: A multi-centre, randomized, controlled pilot study. Mult Scler. 2017;23(10):1394-404.
3. Forsberg A, von Koch L, Nilsagard Y. Effects on balance and walking with the CoDuSe balance exercise program in people with Multiple Sclerosis: A multicenter randomized controlled trial. Mult Scler Int. 2016;2016:7076265.
4. Gleeson M, Sherrington C, Lo S, Keay L. Can the Alexander Technique improve balance and mobility in older adults with visual impairments? A randomized controlled trial. Clin Rehabil. 2015;29(3):244-60.
5. Goodwin VA, Pickering R, Ballinger C, Roberts H, McIntosh E, Lamb S, et al. A multi-centre, randomised controlled trial of the effectiveness of PDSAFE to prevent falls among people with Parkinson's: Study protocol. BMC Neurol. 2015;15:81.
6. Gunn H, Andrade J, Paul L, Miller L, Creanor S, Green C, et al. Balance Right in Multiple Sclerosis (BRiMS): A guided self-management programme to reduce falls and improve quality of life, balance and mobility in people with secondary progressive Multiple Sclerosis: A protocol for a feasibility randomised controlled trial. Pilot Feasibility Stud. 2018;4:26.
7. Gunn H, Andrade J, Paul L, Miller L, Creanor S, Stevens K, et al. A self-management programme to reduce falls and improve safe mobility in people with secondary progressive MS: The BRIMS feasibility RCT. Health Technology Assessment. 2019;23(27):1-165.
8. Gunn H, Stevens KN, Creanor S, Andrade J, Paul L, Miller L, et al. Balance Right in Multiple Sclerosis (BRiMS): a feasibility randomised controlled trial of a falls prevention programme. Pilot Feasibility Stud. 2021;7(1):2.

9. Hatton AL, Dixon J, Rome K, Brauer SG, Williams K, Kerr G. The effects of prolonged wear of textured shoe insoles on gait, foot sensation and proprioception in people with multiple sclerosis: Study protocol for a randomised controlled trial. Trials. 2016;17(1):208.

10. Henderson EJ, Lord SR, Brodie MA, Gaunt DM, Lawrence AD, Close JC, et al. Rivastigmine for gait Stability in Patients with Parkinson's Disease (ReSPonD): A randomised, double-blind, placebo-controlled, phase 2 trial. Lancet Neurol. 2016;15(3):249-58.

11. Hoang P, Schoene D, Gandevia S, Smith S, Lord SR. Effects of a home-based step training programme on balance, stepping, cognition and functional performance in people with multiple sclerosis--A randomized controlled trial. Mult Scler. 2016;22(1):94-103.

12. Ivers R, Coombes J, Sherrington C, Mackean T, Tiedemann A, Hill AM, et al. Healthy ageing among older Aboriginal people: the Ironbark study protocol for a cluster randomised controlled trial. Injury Prevention. 2020;26(6):581-7.

13. Jepsen DB, Ryg J, Jørgensen NR, Hansen S, Masud T. The combined effect of Parathyroid hormone (1-34) and whole-body Vibration exercise in the treatment of OSteoporosis (PaVOS)- study protocol for a randomized controlled trial. Trials. 2018;19(1):186.

14. Khaw KT, Stewart AW, Waayer D, Lawes CMM, Toop L, Camargo CA, Jr., et al. Effect of monthly high-dose vitamin D supplementation on falls and non-vertebral fractures: Secondary and post-hoc outcomes from the randomised, double-blind, placebo-controlled ViDA trial. Lancet Diabetes Endocrinol. 2017;5(6):438-47.

15. Lachance CC, Feldman F, Laing AC, Leung PM, Robinovitch SN, Mackey DC. Study protocol for the Flooring for Injury Prevention (FLIP) Study: A randomised controlled trial in long-term care. Inj Prev. 2016;22(6):453-60.

16. Mansfield A, Aqui A, Centen A, Danells CJ, DePaul VG, Knorr S, et al. Perturbation training to promote safe independent mobility post-stroke: Study protocol for a randomized controlled trial. BMC Neurol. 2015;15:87.

17. Martini DN, Zeeboer E, Hildebrand A, Fling BW, Hugos CL, Cameron MH. ADSTEP: Preliminary investigation of a multicomponent walking aid program in people with Multiple Sclerosis. Arch Phys Med Rehabil. 2018;99(10):2050-8.

18. Matthews MJ, Yusuf M, Doyle C, Thompson C. Quadrupedal movement training improves markers of cognition and joint repositioning. Hum Mov Sci. 2016;47:70-80.

19. Morone G, Annicchiarico R, Iosa M, Federici A, Paolucci S, Cortes U, et al. Overground walking training with the i-Walker, a robotic servo-assistive device, enhances balance in patients with subacute stroke: A randomized controlled trial. J Neuroeng Rehabil. 2016;13(1):47.

20. Morris ME, Menz HB, McGinley JL, Watts JJ, Huxham FE, Murphy AT, et al. A randomized controlled trial to reduce falls in people with Parkinson's Disease. Neurorehabil Neural Repair. 2015;29(8):777-85.

21. Nyman SR, Hayward C, Ingram W, Thomas P, Thomas S, Vassallo M, et al. A randomised controlled trial comparing the effectiveness of Tai Chi alongside usual care with usual care alone on the postural balance of community-dwelling people with dementia: protocol for the TACIT trial (TAi ChI for people with demenTia). BMC Geriatr. 2018;18(1):263.

22. Rose DK, DeMark L, Fox EJ, Clark DJ, Wludyka P. A backward walking training program to improve balance and mobility in acute stroke: A pilot randomized controlled trial. J Neurol Phys Ther. 2018;42(1):12-21.

23. Taylor PN, Sampson T, Beare B, Donavon-Hall M, Thomas PW, Marques E, et al. The effectiveness of peroneal nerve functional electrical simulation for the reduction of bradykinesia

in Parkinson's disease: A feasibility study for a randomised control trial. Clinical Rehabilitation. 2021;35(4):546-57.

24. Walker GM, Armstrong S, Gordon AL, Gladman J, Robertson K, Ward M, et al. The Falls in Care Home study: A feasibility randomized controlled trial of the use of a risk assessment and decision support tool to prevent falls in care homes. Clin Rehabil. 2016;30(10):972-83.

Articles excluded because not examining effectiveness, cost-effectiveness, or safety of interventions to prevent and/or manage falls (n=18)

1. Barzideh A, Marzolini S, Danells C, Jagroop D, Huntley AH, Inness EL, et al. Effect of reactive balance training on physical fitness poststroke: Study protocol for a randomised non-inferiority trial. BMJ Open. 2020;10(6):e035740.

2. Becker C, Lord SR, Studenski SA, Warden SJ, Fielding RA, Recknor CP, et al. Myostatin antibody (LY2495655) in older weak fallers: A proof-of-concept, randomised, phase 2 trial. Lancet Diabetes Endocrinol. 2015;3(12):948-57.

3. Bongartz M, Kiss R, Ullrich P, Eckert T, Bauer J, Hauer K. Development of a home-based training program for post-ward geriatric rehabilitation patients with cognitive impairment: Study protocol of a randomized-controlled trail. BMC Geriatr. 2017;17(1):214.

4. Cavalcante BR, De Souza MF, Liu-Ambrose T, Behm D, Pitangui ACR, De Araújo RC. Effects of resistance exercise with instability on neurocognitive functions (REI STUDY): Study protocol for a proof-of-concept clinical trial in older adults with subjective cognitive complaints. Motriz Revista de Educacao Fisica. 2019;25(2).

5. Hinrichs T, Bucker B, Wilm S, Klaassen-Mielke R, Brach M, Platen P, et al. Adverse events in mobility-limited and chronically ill elderly adults participating in an exercise intervention study supported by general practitioner practices. J Am Geriatr Soc. 2015;63(2):258-69.

6. Kaut O, Brenig D, Marek M, Allert N, Wullner U. Postural stability in Parkinson's disease patients is improved after stochastic resonance therapy. Parkinsons Dis. 2016;2016:7948721.
7. Lamb SE, Mistry D, Alleyne S, Atherton N, Brown D, Copsey B, et al. Aerobic and strength training exercise programme for cognitive impairment in people with mild to moderate dementia: The DAPA RCT. Health Technology Assessment. 2018;22(28):1-201.
8. Marian IR, Williamson E, Garrett A, Lamb SE, Dutton SJ. Better Outcomes for Older people with Spinal Trouble (BOOST) trial: Statistical analysis plan for a randomised controlled trial of a combined physical and psychological intervention for older adults with neurogenic claudication. Trials. 2020;21(1):667.
9. Meeks S, Ludwin BM, Looney SW. Falls as adverse events in psychosocial treatment of depression: Findings from a clinical trial in nursing homes. Contemp Clin Trials Commun. 2016;3:139-41.
10. Morat T, Mechling H. Training in the functional movement circle to promote strength and mobility-related activities in older adults: Arandomized controlled trial. Eur J Ageing. 2015;12(2):105-18.
11. Patil R, Kolu P, Raitanen J, Valvanne J, Kannus P, Karinkanta S, et al. Cost-effectiveness of vitamin D supplementation and exercise in preventing injurious falls among older home-dwelling women: Findings from an RCT. Osteoporos Int. 2016;27(1):193-201.
12. Reid KF, Laussen J, Bhatia K, Englund DA, Kirn DR, Price LL, et al. Translating the Lifestyle Interventions and Independence for Elders clinical trial to older adults in a real-world community-based setting. J Gerontol A Biol Sci Med Sci. 2019;74(6):924-8.

13. Said CM, Morris ME, McGinley JL, Szoeke C, Workman B, Liew D, et al. Additional structured physical activity does not improve walking in older people (> 60 years) undergoing inpatient rehabilitation: A randomised trial. Journal of Physiotherapy. 2018;64(4):237-44.

14. Salbach NM, Barclay R, Webber SC, Jones CA, Mayo NE, Lix LM, et al. A theory-based, task-oriented, outdoor walking programme for older adults with difficulty walking outdoors: Protocol for the Getting Older Adults Outdoors (GO-OUT) randomised controlled trial. BMJ Open. 2019;9(4):e029393.

15. Teh R, Kerse N, Waters DL, Hale L, Pillai A, Leilua E, et al. Study protocol of a randomised controlled trial to examine the impact of a complex intervention in pre-frail older adults. Aging Clinical and Experimental Research. 2019;31(10):1407-17.

16. Walters K, Frost R, Kharicha K, Avgerinou C, Gardner B, Ricciardi F, et al. Home-based health promotion for older people with mild frailty: The HomeHealth intervention development and feasibility RCT. Health Technol Assess. 2017;21(73):1-128.

17. Williamson E, Ward L, Vadher K, Dutton SJ, Parker B, Petrou S, et al. Better Outcomes for Older people with Spinal Trouble (BOOST) Trial: A randomised controlled trial of a combined physical and psychological intervention for older adults with neurogenic claudication, a protocol. BMJ Open. 2018;8(10):e022205.

18. Witham MD, Lees JS, White M, Band M, Bell S, Chantler DJ, et al. Vitamin K supplementation to improve vascular stiffness in CKD: The K4Kidneys randomized controlled trial. J Am Soc Nephrol. 2020;31(10):2434-45.

Articles excluded because protocol, pilot or feasibility study (n=44)

1. Arkkukangas M, Johnson ST, Hellstrom K, Soderlund A, Eriksson S, Johansson AC. A feasibility study of a randomised controlled trial comparing fall prevention using exercise with or

without the support of motivational interviewing. Prev Med Rep. 2015;2:134-40.

2. Barban F, Annicchiarico R, Federici A, Mazzù I, Lombardi MG, Giuli S, et al. ICT solutions to develop an effective motor and cognitive training to reduce risk of falls - The I-DONT-FALL Project. In Proceedings of the 1st International Conference on Information and Communication Technologies for Ageing Well and e-Health, ICT4AgeingWell. 2015: 259-263. DOI: 10.5220/0005490802590263.

3. Barker AL, Cameron PA, Hill KD, Flicker L, Haines TP, Lowthian JA, et al. RESPOND--A patient-centred programme to prevent secondary falls in older people presenting to the emergency department with a fall: Protocol for a multicentre randomised controlled trial. Inj Prev. 2015;21(1):e1.

4. Bhasin S, Gill TM, Reuben DB, Latham NK, Gurwitz JH, Dykes P, et al. Strategies to reduce injuries and develop confidence in elders (STRIDE): A cluster-randomized pragmatic trial of a multifactorial fall injury prevention strategy: Design and methods. Journals of Gerontology - Series A Biological Sciences and Medical Sciences. 2018;73(8):1053-61.

5. Bjerk M, Brovold T, Skelton DA, Bergland A. A falls prevention programme to improve quality of life, physical function and falls efficacy in older people receiving home help services: Study protocol for a randomised controlled trial. BMC Health Serv Res. 2017;17(1):559.

6. Bruce J, Lall R, Withers EJ, Finnegan S, Underwood M, Hulme C, et al. A cluster randomised controlled trial of advice, exercise or multifactorial assessment to prevent falls and fractures in community-dwelling older adults: Protocol for the prevention of falls injury trial (PreFIT). BMJ Open. 2016;6(1):e009362.

7. Callisaya ML, Jayakody O, Vaidya A, Srikanth V, Farrow M, Delbaere K. A novel cognitive-motor exercise program delivered via a tablet to improve mobility in older people with cognitive impairment – StandingTall Cognition and Mobility. Experimental Gerontology. 2021;152.

8. Cockayne S, Pighills A, Adamson J, Fairhurst C, Drummond A, Hewitt C, et al. Can occupational therapist-led home environmental assessment prevent falls in older people? A modified cohort randomised controlled trial protocol. BMJ Open. 2018;8(9):e022488.

9. Dautel A, Eckert T, Gross M, Hauer K, Schaüfele M, Lacroix A, et al. Multifactorial intervention for hip and pelvic fracture patients with mild to moderate cognitive impairment: Study protocol of a dual-centre randomised controlled trial (OF-CARE). BMC Geriatrics. 2019;19(1).

10. Delbaere K, Valenzuela T, Woodbury A, Davies T, Yeong J, Steffens D, et al. Evaluating the effectiveness of a home-based exercise programme delivered through a tablet computer for preventing falls in older community-dwelling people over 2 years: Study protocol for the Standing Tall randomised controlled trial. BMJ Open. 2015;5(10):e009173.

11. Fischbacher M, Chocano-Bedoya PO, Meyer U, Bopp I, Mattle M, Kressig RW, et al. Safety and feasibility of a Dalcroze eurhythmics and a simple home exercise program among older adults with mild cognitive impairment (MCI) or mild dementia: the MOVE for your MIND pilot trial. Pilot Feasibility Stud. 2020;6:101.

12. Ganz DA, Siu AL, Magaziner J, Latham NK, Travison TG, Lorenze NP, et al. Protocol for serious fall injury adjudication in the Strategies to Reduce Injuries and Develop Confidence in Elders (STRIDE) study. Injury Epidemiology. 2019;6(1).

13. Giangregorio LM, Gibbs JC, Templeton JA, Adachi JD, Ashe MC, Bleakney RR, et al. Build better bones with exercise (B3E pilot trial): Results of a feasibility study of a multicenter

randomized controlled trial of 12 months of home exercise in older women with vertebral fracture. Osteoporos Int. 2018;29(11):2545-56.

14. Granbom M, Clemson L, Roberts L, Hladek MD, Okoye SM, Liu M, et al. Preventing falls among older fallers: Study protocol for a two-phase pilot study of the multicomponent LIVE LiFE program. Trials. 2019;20(1).

15. Harwood RH, van der Wardt V, Goldberg SE, Kearney F, Logan P, Hood-Moore V, et al. A development study and randomised feasibility trial of a tailored intervention to improve activity and reduce falls in older adults with mild cognitive impairment and mild dementia. Pilot Feasibility Stud. 2018;4:49.

16. Hawley-Hague H, Tacconi C, Mellone S, Martinez E, Easdon A, Yang FB, et al. Can smartphone technology be used to support an effective home exercise intervention to prevent falls amongst community dwelling older adults?: the TOGETHER feasibility RCT study protocol. BMJ Open. 2019;9(9):e028100.

17. Hill AM, Etherton-Beer C, McPhail SM, Morris ME, Flicker L, Shorr R, et al. Reducing falls after hospital discharge: A protocol for a randomised controlled trial evaluating an individualised multimodal falls education programme for older adults. BMJ Open. 2017;7(2):e013931.

18. Jansen CP, Nerz C, Kramer F, Labudek S, Klenk J, Dams J, et al. Comparison of a group-delivered and individually delivered lifestyle-integrated functional exercise (LiFE) program in older persons: A randomized noninferiority trial. BMC Geriatr. 2018;18(1):267.

19. Johansson E, Jonsson H, Dahlberg R, Patomella AH. The efficacy of a multifactorial falls-prevention programme, implemented in primary health care. British Journal of Occupational Therapy. 2018;81(8):474-81.

20. Keall M, Tupara H, Pierse N, Wilkie M, Baker M, Howden-Chapman P, et al. Study protocol of a randomized controlled trial of home modification to prevent home fall injuries in houses with Māori occupants. Methods Protoc. 2020;3(4).

21. Keay L, Dillon L, Clemson L, Tiedemann A, Sherrington C, McCluskey P, et al. PrevenTing Falls in a high-risk, vision-impaired population through specialist ORientation and Mobility services: Protocol for the PlaTFORM randomised trial. Injury Prevention. 2018;24(6):459-66.

22. Keogh JW, Henwood T, Gardiner P, Tuckett A, Hodgkinson B, Rouse K. Examining evidence based resistance plus balance training in community-dwelling older adults with complex health care needs: Trial protocol for the Muscling Up Against Disability project. Arch Gerontol Geriatr. 2017;68:97-105.

23. Kienle GS, Werthmann PG, Grotejohann B, Kaier K, Steinbrenner I, Voigt-Radloff S, et al. A multi-centre, parallel-group, randomised controlled trial to assess the efficacy and safety of eurythmy therapy and Tai Chi in comparison with standard care in chronically ill elderly patients with increased risk of falling (ENTAiER): A trial protocol. BMC Geriatr. 2020;20(1):108.

24. Lark SD, Wadsworth DP. Physiological, psychological and functional changes with whole body vibration exercise in the elderly: FEVER methodology and protocols. Contemp Clin Trials. 2015;44:129-33.

25. Martin T, Weatherall M, Anderson TJ, MacAskill MR. A randomized controlled feasibility trial of a specific cueing program for falls management in persons with Parkinson disease and freezing of gait. J Neurol Phys Ther. 2015;39(3):179-84.

26. Michos ED, Mitchell CM, Miller ER, III, Sternberg AL, Juraschek SP, Schrack JA, et al. Rationale and design of the Study To Understand Fall Reduction and Vitamin D in You

(STURDY): A randomized clinical trial of Vitamin D supplement doses for the prevention of falls in older adults. Contemporary Clinical Trials. 2018;73:111-22.

27. Oliveira JS, Sherrington C, Lord S, Sesto R, Youkhana S, Camara GC, et al. Yoga-based exercise to prevent falls in community-dwelling people aged 60 years and over: Study protocol for the Successful AGEing (SAGE) yoga randomised controlled trial. BMJ Open Sport Exerc Med. 2020;6(1):e000878.

28. Pettersson B, Lundin-Olsson L, Skelton DA, Liv P, Zingmark M, Rosendahl E, et al. Effectiveness of a self-managed digital exercise programme to prevent falls in older community-dwelling adults: Study protocol for the Safe Step randomised controlled trial. BMJ Open. 2020;10(5):e036194.

29. Renehan E, Meyer C, Elliott RA, Batchelor F, Said C, Haines T, et al. Posthospital falls prevention intervention: A mixed-methods study. Journal of Aging and Physical Activity. 2019;27(2):155-65.

30. Said CM, Morris ME, McGinley JL, Szoeke C, Workman B, Liew D, et al. Evaluating the effects of increasing physical activity to optimize rehabilitation outcomes in hospitalized older adults (MOVE Trial): Study protocol for a randomized controlled trial. Trials. 2015;16:13.

31. Schoon Y, Bongers KTJ, Olde Rikkert MGM. Feasibility study by a single-blind randomized controlled trial of self-management of mobility with a gait-speed feedback device by older persons at risk for falling. Assistive Technology. 2020;32(4):222-8.

32. Skelton DA, Bailey C, Howel D, Cattan M, Deary V, Coe D, et al. Visually Impaired OLder people's Exercise programme for falls prevenTion (VIOLET): A feasibility study protocol. BMJ Open. 2016;6(8):e011996.

33. Stark S, Somerville E, Keglovits M, Conte J, Li M, Hu YL, et al. Protocol for the home hazards removal program (HARP) study: A pragmatic, randomized clinical trial and implementation study. BMC Geriatr. 2017;17(1):90.

34. Sturnieks DL, Menant J, Valenzuela M, Delbaere K, Sherrington C, Herbert RD, et al. Effect of cognitive-only and cognitive-motor training on preventing falls in community-dwelling older people: Protocol for the smart±step randomised controlled trial. BMJ Open. 2019;9(8):e029409.

35. Taylor NF, O'Halloran PD, Watts JJ, Morris R, Peiris CL, Porter J, et al. Motivational interviewing with community-dwelling older adults after hip fracture (MIHip): Protocol for a randomised controlled trial. BMJ Open. 2021;11(6).

36. Tiedemann A, Rissel C, Howard K, Tong A, Merom D, Smith S, et al. Health coaching and pedometers to enhance physical activity and prevent falls in community-dwelling people aged 60 years and over: Study protocol for the Coaching for Healthy AGEing (CHAnGE) cluster randomised controlled trial. BMJ Open. 2016;6(5):e012277.

37. van Gameren M, Bossen D, Bosmans JE, Visser B, Frazer SWT, Pijnappels M. The (cost-) effectiveness of an implemented fall prevention intervention on falls and fall-related injuries among community-dwelling older adults with an increased risk of falls: Protocol for the in balance randomized controlled trial. BMC Geriatr. 2021;21(1):381.

38. van Schooten KS, Callisaya ML, O'Dea B, Lung T, Anstey K, Lord SR, et al. Protocol of a 12-month multifactorial eHealth programme targeting balance, dual-tasking and mood to prevent falls in older people: the StandingTall+ randomised controlled trial. BMJ Open. 2021;11(4):e051085.

39. Waterman H, Ballinger C, Brundle C, Chastin S, Gage H, Harper R, et al. A feasibility study to prevent falls in older people who are sight impaired: The VIP2UK randomised controlled trial. Trials. 2016;17(1):464.

40. Whitney J, Jackson SHD, Martin FC. Feasibility and efficacy of a multi-factorial intervention to prevent falls in older adults with cognitive impairment living in residential care (ProF-Cog). A feasibility and pilot cluster randomised controlled trial. BMC Geriatr. 2017;17(1):115.

41. Wildes TS, Winter AC, Maybrier HR, Mickle AM, Lenze EJ, Stark S, et al. Protocol for the Electroencephalography Guidance of Anesthesia to Alleviate Geriatric Syndromes (ENGAGES) study: A pragmatic, randomised clinical trial. BMJ Open. 2016;6(6):e011505.

42. Witham MD, Price RJG, Band MM, Hannah MS, Fulton RL, Clarke CL, et al. Effect of Vitamin K2 on postural sway in older people who fall: A randomized controlled trial. Journal of the American Geriatrics Society. 2019;67(10):2102-7.

43. Wylie G, Menz HB, McFarlane S, Ogston S, Sullivan F, Williams B, et al. Podiatry intervention versus usual care to prevent falls in care homes: Pilot randomised controlled trial (the PIRFECT study). BMC Geriatr. 2017;17(1):143.

44. Young HML, March DS, Highton PJ, Graham-Brown MPM, Churchward DC, Grantham C, et al. Exercise for people living with frailty and receiving haemodialysis: A mixed-methods randomised controlled feasibility study. BMJ Open. 2020;10(11):e041227.

Articles excluded because duplicate report of already included RCT (n=5)

1. Bruce J, Hossain A, Lall R, Withers EJ, Finnegan S, Underwood M, et al. Fall prevention interventions in primary care to reduce fractures and falls in people aged 70 years and over: The PreFIT three-arm cluster RCT. Health Technology Assessment. 2021;25(34):1-+.

2. Cockayne S, Rodgers S, Green L, Fairhurst C, Adamson J, Scantlebury A, et al. Clinical effectiveness and cost-effectiveness of a multifaceted podiatry intervention for falls prevention in older people: A multicentre cohort randomised controlled trial (the REducing Falls with ORthoses and a Multifaceted podiatry intervention trial). Health Technol Assess. 2017;21(24):1-198.

3. Corbacho B, Cockayne S, Fairhurst C, Hewitt CE, Hicks K, Kenan AM, et al. Cost-effectiveness of a multifaceted podiatry intervention for the prevention of falls in older people: The REducing Falls with ORthoses and a Multifaceted podiatry intervention trial findings. Gerontology. 2018;64(5):503-12.

4. Scragg RKR. Overview of results from the Vitamin D Assessment (ViDA) study. J Endocrinol Invest. 2019;42(12):1391-9.

5. Snooks HA, Anthony R, Chatters R, Dale J, Fothergill RT, Gaze S, et al. Paramedic assessment of older adults after falls, including community care referral pathway: Cluster randomized trial. Ann Emerg Med. 2017;70(4):495-505 e28.
